# Supplementary material for: Global analysis of protein arginine methylation
Source: Cell Rep Methods. 2021 Jun 21;1(2):100016. doi: 10.1016/j.crmeth.2021.100016 (PMC9017121; doi:10.1016/j.crmeth.2021.100016)
Supplement: Document S1. Figures S1–S4 and Table S1 [file mmc1.pdf]

Cell Reports Methods, Volume *1*

Supplemental information

Global analysis of protein arginine methylation

Fangrong Zhang, Jakob Kerbl-Knapp, Maria J. Rodriguez Colman, Andreas Meinitzer, Therese Macher, Nemanja Vujić, Sandra Fasching, Evelyne Jany-Luig, Melanie Korbelius, Katharina B. Kuentzel, Maximilian Mack, Alena Akhmetshina, Anita Pirchheim, Margret Paar, Beate Rinner, Gerd Hörl, Ernst Steyrer, Ulrich Stelzl, Boudewijn Burgering, Tobias Eisenberg, Brigitte Pertschy, Dagmar Kratky, and Tobias Madl

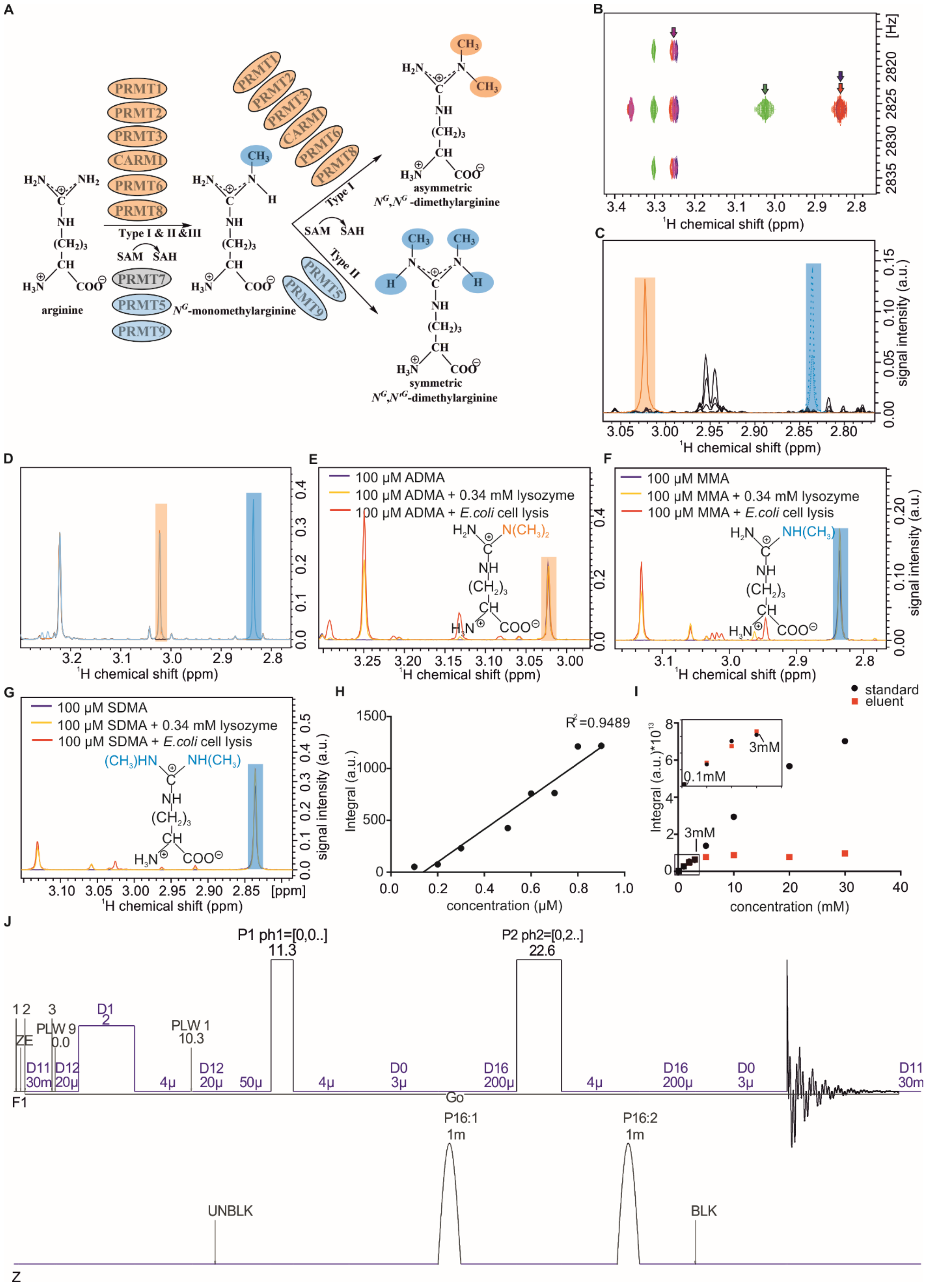
A
PRMT1
PRMT2
PRMT3
CARM1
PRMT6
PRMT8
Type I & II &III
SAM SAH
PRMT7
PRMT5
PRMT9
arginine
N^G-monomethylarginine
Type I
Type II
SAM SAH
asymmetric
N^G,N^G-dimethylarginine
symmetric
N^G,N'^G-dimethylarginine
B
[Hz]
^{1}H chemical shift (ppm)
C
signal intensity (a.u.)
^{1}H chemical shift (ppm)
D
^{1}H chemical shift (ppm)
E
100 μM ADMA
100 μM ADMA + 0.34 mM lysozyme
100 μM ADMA + E.coli cell lysis
^{1}H chemical shift (ppm)
F
100 μM MMA
100 μM MMA + 0.34 mM lysozyme
100 μM MMA + E.coli cell lysis
signal intensity (a.u.)
^{1}H chemical shift (ppm)
G
100 μM SDMA
100 μM SDMA + 0.34 mM lysozyme
100 μM SDMA + E.coli cell lysis
signal intensity (a.u.)
^{1}H chemical shift (ppm)
[ppm]
H
R^2=0.9489
Integral (a.u.)
concentration (μM)
I
standard
eluent
Integral (a.u.)*10^{13}
3mM
0.1mM
concentration (mM)
J
P1 ph1=[0,0..]
11.3
P2 ph2=[0,2..]
22.6
ZE
PLW 9
0.0
D1
2
PLW 1
10.3
D11
30m
D12
20μ
4μ
50μ
D0
3μ
D16
200μ
F1
Go
P16:1
1m
P16:2
1m
UNBLK
BLK
Z

Figure S1. Related to Figure 1. Absolute quantification of protein arginine methylation by ArgMet-NMR.

(A) Arginine residues are methylated by the protein arginine methyltransferase (PRMT) (type I, orange; type II, blue; type III, grey) family members. PRMTs catalyze either the formation of monomethylarginine (MMA) (blue), asymmetric dimethylarginine (ADMA) (orange) or symmetric dimethylarginine (SDMA) (blue).
(B) Overlay of ^{1}H 2D J-resolved experiments of arginine (magenta), ADMA (green), MMA (purple) and SDMA (red), each 100 μM.
(C) Overlay of ^{1}H 1D projections of 2D J-resolved, virtually decoupled NMR spectra of *E. coli* lysates spiked with 100 μM ADMA, MMA and SDMA (black, solid line) before whole workflow, and 100 μM ADMA (orange, solid line), MMA (blue, dotted line) or SDMA (blue, dashed line), respectively, showing free methylarginines are negligible using our protocol.
(D) Overlay of 1H 1D projections of 2D J-resolved, virtually decoupled NMR spectra of metabolites extraction from HeLa cells (black), and then spiked with 100 μM ADMA (orange), MMA and SDMA (blue), respectively.
(E) Overlay of ^{1}H 1D projections of 2D J-resolved, virtually decoupled NMR spectra of ADMA recovery from lysozyme (yellow) and *E. coli* cell lysates (red). Shaded regions represent characteristic regions of ADMA (orange) methyl groups.
(F) Overlay of ^{1}H 1D projections of 2D J-resolved, virtually decoupled NMR spectra of MMA recovery from lysozyme (yellow) and *E. coli* cell lysates (red). Shaded regions represent characteristic regions of MMA (blue) methyl groups.
(G) Overlay of ^{1}H 1D projections of 2D J-resolved, virtually decoupled NMR spectra of SDMA recovery from lysozyme (yellow) and *E. coli* cell lysates (red). Shaded regions represent characteristic regions of SDMA (blue) methyl groups.
(H) Correlation between integrals and concentration changes of ADMA signal of ^{1}H 1D projections of 2D J-resolved spectra. Correlation coefficient (R^2 = 0. 9489, p = 0.033) was computed with the Pearson Product Moment statistic.
(I) Integrals of arginine (red, square) recovery from SPE compared to standard concentrations (black, circle).
(J) Pulse sequence of the 2D JRES NMR experiment.

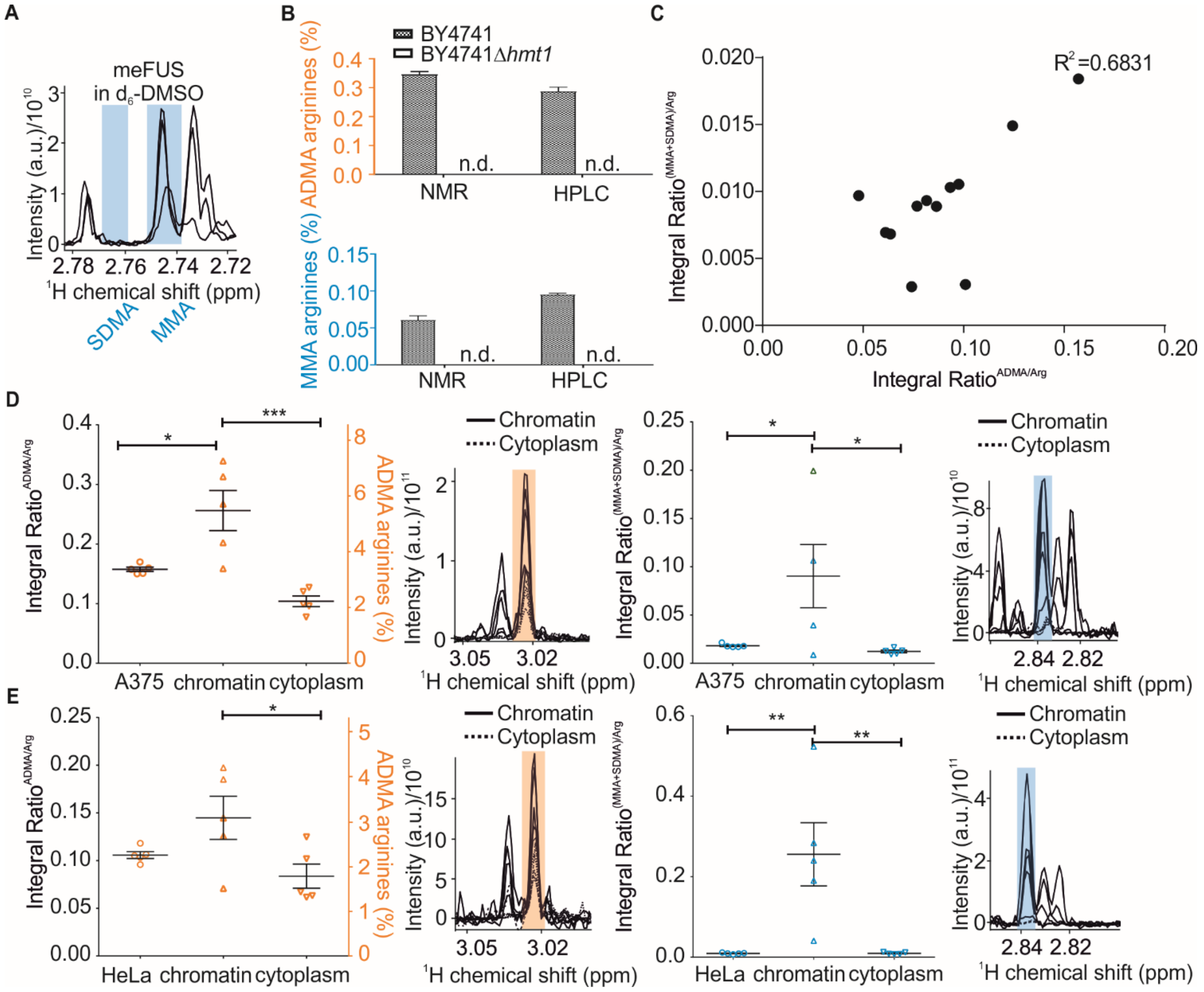

Figure S2. Related to Figure 2. Characterisation of ArgMet in purified proteins, yeast and mammalian cell lysates.

(A) Spectral overlays of characteristic MMA and SDMA NMR methyl signals in d_6-DMSO (n=3). Shaded regions represent characteristic regions of MMA and SDMA (blue) methyl groups.

(B) HPLC and NMR based protein ArgMet quantification of yeast lysates (n=3; mean ± SD; n.d. - not detectable). ADMA or MMA levels in relation to the total amount of arginine are indicated. The MMA peak obtained from HPLC showed spectral overlap interference.

(C) The correlation between integral ratios of ADMA/arginine and (SDMA/MMA)/arginine. Correlation coefficient (R^2 = 0. 6381, p = 0.0143) was computed with the Pearson Product Moment statistic.

(D) ArgMet quantification of chromatin and cytoplasm in A375 cells (n=5; mean ± SD; *p < 0.05, **p < 0.01, ***p < 0.001, ****p < 0.0001). Spectral overlays of characteristic ADMA (orange) and MMA/SDMA (blue), NMR methyl signals are shown as shaded regions.

(E) ArgMet quantification of chromatin and cytoplasm in HeLa cells (n=5; mean ± SD; *p < 0.05, **p < 0.01, ***p < 0.001, ****p < 0.0001). Spectral overlays of characteristic ADMA (orange) and MMA/SDMA (blue), NMR methyl signals are shown as shaded regions.

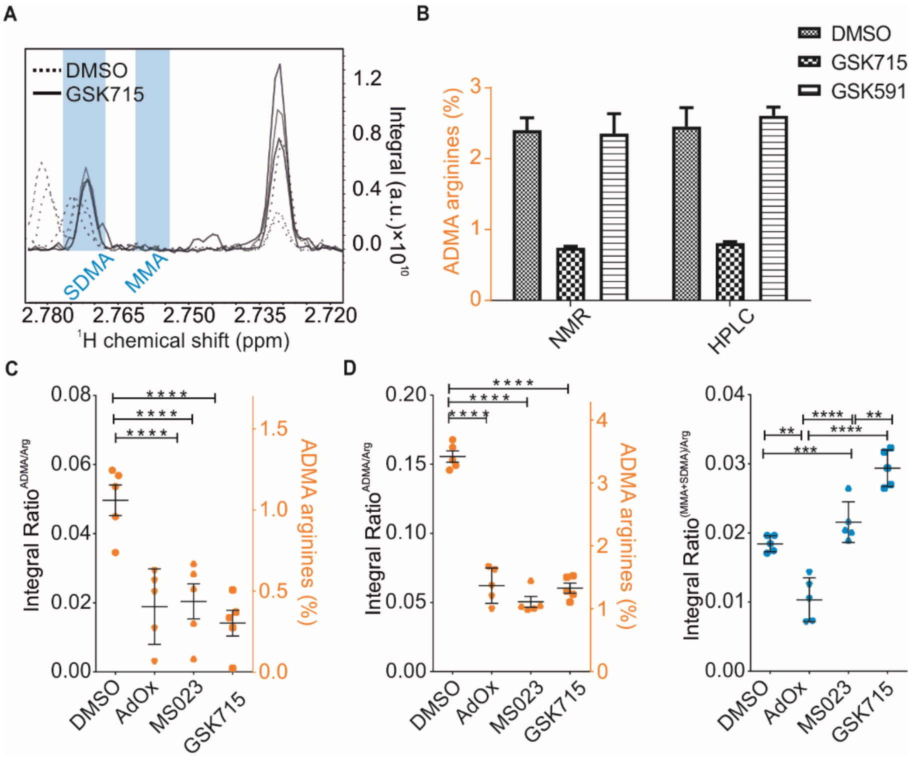

Figure S3. Related to Figure 3. ArgMet NMR enables quantification of protein ArgMet modulation and dynamics.

(A) Spectral overlays of characteristic MMA and SDMA NMR methyl signals in d_6-DMSO show that MMA and SDMA methyl resonances can be resolved (n=3), and higher SDMA represented in GSK715 than DMSO. Shaded regions represent characteristic regions of MMA and SDMA (blue) methyl groups.

(B) HPLC and NMR based protein ArgMet quantification of HeLa cells treated for 3 days with either DMSO, 2 µM GSK3368715 (GSK715) or 1 µM GSK3203591 (GSK591) (n=3; mean ± SD). ADMA levels in relation to the total amount of arginine are indicated.

(C) Protein ArgMet quantification of fibroblast cells treated for 3 days with either DMSO, 40 µM AdOx, 10 µM MS023, 2 µM GSK715 (n=5; mean ± SD; *p < 0.05, **p < 0.01, ***p < 0.001, ****p < 0.0001). ADMA levels with respect to total amounts of arginine are shown. Both MMA and SDMA are non-detectable in these conditions.

(D) Protein ArgMet quantification of A375 cells treated for 3 days with either DMSO, 40 µM AdOx, 10 µM MS023, 2 µM GSK715 (n=5; mean ± SD; *p < 0.05, **p < 0.01, ***p < 0.001, ****p < 0.0001). ADMA levels are presented with respect to total amounts of arginine.

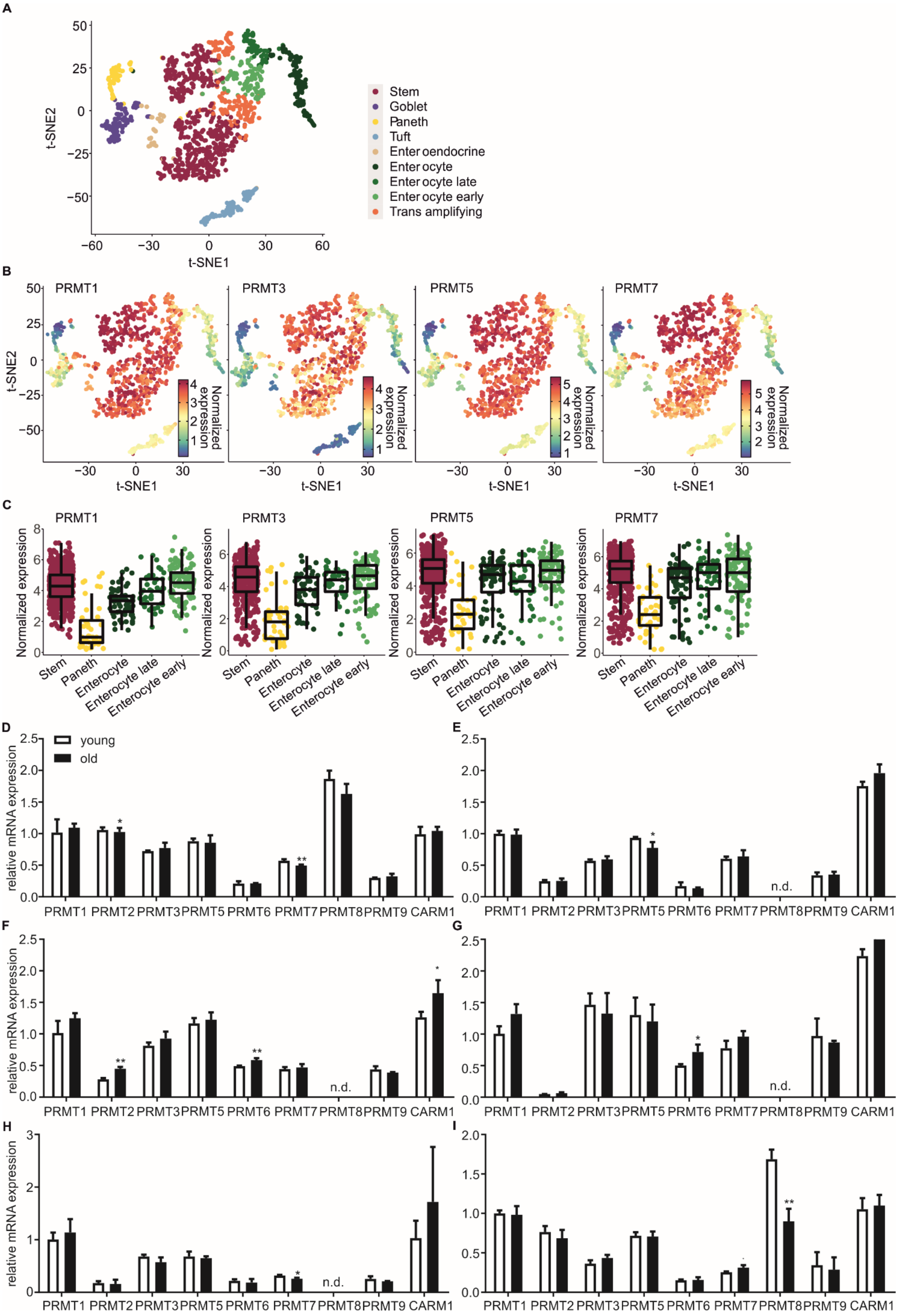
A
t-SNE2
t-SNE1
Stem
Goblet
Paneth
Tuft
Enter oendocrine
Enter ocyte
Enter ocyte late
Enter ocyte early
Trans amplifying
B
PRMT1
PRMT3
PRMT5
PRMT7
Normalized expression
C
Normalized expression
Stem
Paneth
Enterocyte
Enterocyte late
Enterocyte early
D
E
F
G
H
I
young
old
relative mRNA expression
PRMT1 PRMT2 PRMT3 PRMT5 PRMT6 PRMT7 PRMT8 PRMT9 CARM1
n.d.

Figure S4. Related to Figure 4. NMR enables characterization of ArgMet in cell differentiation and ageing *in vivo*.

(A) t-distributed stochastic neighbourhood embedding (t-SNE) visualization of 1,522 single-cell full-length sc-RNaseq data. Cluster annotation was done based on the expression of known cell type markers.
(B) PRMT1, 3, 5 and 7 gene expression levels are plotted on t-SNE plots.
(C) Boxplots represent the quantification of gene expression in each cluster gene (median values and 25th and 75th percentiles). The analysis was performed on mouse small intestine single cell RNAseq data set from Haber *et al.* (Haber et al., 2017) and the analysis was performed as described in Ludikhuize *et al.* (Ludikhuize et al., 2020).
(D-I) mRNA expression of PRMTs in (D) brain, (E) heart, (F) kidney, (G) liver, (H) spleen and (I) lung from young (9-11 weeks) and old mice (96-104 weeks) analysed by real-time PCR and normalized to cyclophilin A as reference gene. Expression profiles and associated statistical parameters were determined by the $2^{-\Delta\Delta Ct}$ method. Data represent means ± SD (n=3); *$p < 0.05$, **$p < 0.01$, ***$p < 0.001$.

**Table S1. List of oligonucleotides**		
Name	Sequence	Note
PRMT1 forward primer	GCCCGCAAGGTTATTGGGAT	real-time PCR
PRMT1 reverse primer	TGGTCTAACTTGTTGGCTTTGAC	real-time PCR
PRMT2 forward primer	AGCGCCGAGAAAGACTACC	real-time PCR
PRMT2 reverse primer	GGCCTTGAAAAGAACTCCTTGA	real-time PCR
PRMT3 forward primer	GGGATCGGTCTACCCTGACAT	real-time PCR
PRMT3 reverse primer	AGCTTCCGGTATAACTGCTTTC	real-time PCR
PRMT5 forward primer	GGAACTCTGAAGCGGCTATGT	real-time PCR
PRMT5 reverse primer	CTTCCTGATTAAGGGGCAATAGG	real-time PCR
PRMT6 forward primer	GATGGGCTACGGACTTCTGC	real-time PCR
PRMT6 reverse primer	GCATCTGGTCGCTAATCGGG	real-time PCR
PRMT7 forward primer	GCCAGGTCATCCTATGCCG	real-time PCR
PRMT7 reverse primer	GCCAATGTCAAGAACCAAGGC	real-time PCR
PRMT8 forward primer	ACGTGGTAGCAATCGAAGACA	real-time PCR
PRMT8 reverse primer	GCTCCTTCATGGCAACATCC	real-time PCR
PRMT9 forward primer	CAGAAACTGTCGATGCAGGTG	real-time PCR
PRMT9 reverse primer	AGCACCAACCCTATGATGCC	real-time PCR
CARM1 forward primer	ATCGCCCTCTACAGCCATGA	real-time PCR
CARM1 reverse primer	CTGTCTGCCCACACGACTG	real-time PCR

Table S1. Related to STAR Methods "RNA isolation, reverse transcription and real-time PCR" Key Resources Table subheading "Oligonucleotides". Shown are all oligonucleotides used in this study for real-time PCR.
