# Supplementary material for: Global analysis of protein arginine methylation
Source: Cell Rep Methods. 2021 Jun 21;1(2):100016. doi: 10.1016/j.crmeth.2021.100016 (PMC9017121; doi:10.1016/j.crmeth.2021.100016)
Supplement: Document S2. Article plus supplemental information [file mmc2.pdf]

Cell Reports
Methods

Article

Global analysis of protein arginine methylation

Graphical abstract

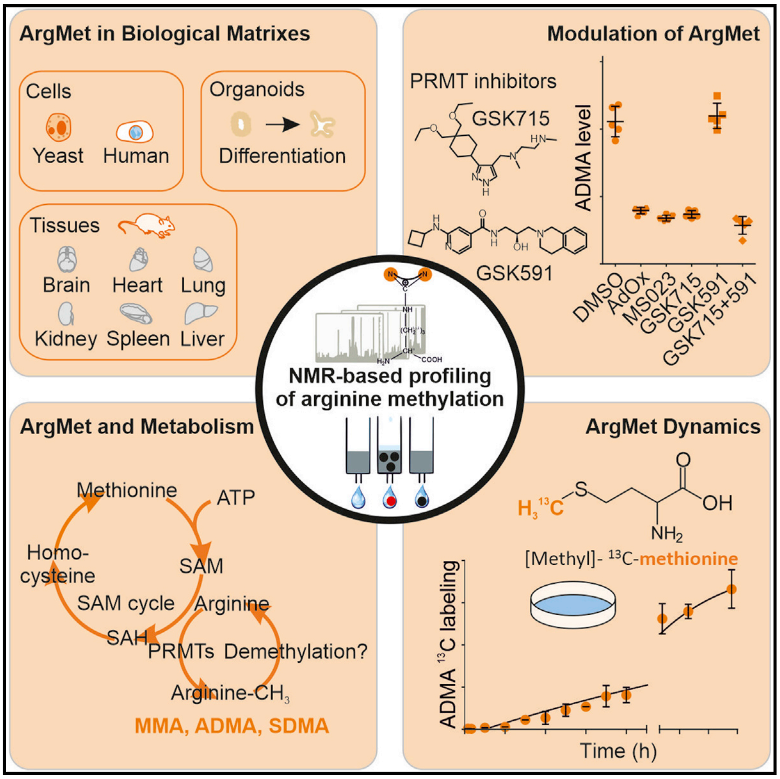

Authors

Fangrong Zhang, Jakob Kerbl-Knapp, Maria J. Rodriguez Colman, ..., Brigitte Pertschy, Dagmar Kratky, Tobias Madl

Correspondence

tobias.madl@medunigraz.at

In brief

Zhang et al. report an NMR-based strategy for the quantification of protein arginine methylation in biological samples. The method provides routes for studying modulation of protein arginine methylation by inhibitors, metabolites, and biological processes.

Highlights

- NMR enables robust quantification of (methylated) arginines in biological matrices
- Arginine methylation represents a highly abundant post-translational modification
- Arginine methylation can be modulated by small-molecule inhibitors and metabolites
- Alterations provide insights into phenotypes of cancer, differentiation, and aging

Zhang et al., 2021, Cell Reports Methods *1*, 100016
June 21, 2021 © 2021 The Author(s).
https://doi.org/10.1016/j.crmeth.2021.100016

CellPress

Cell Reports Methods

CellPress
OPEN ACCESS

Article

Global analysis of protein arginine methylation

Fangrong Zhang,[1] Jakob Kerbl-Knapp,[1] Maria J. Rodriguez Colman,[2] Andreas Meinitzer,[3] Therese Macher,[1] Nemanja Vujić,[1,4] Sandra Fasching,[5] Evelyne Jany-Luig,[5] Melanie Korbelius,[1] Katharina B. Kuentzel,[1] Maximilian Mack,[4,6] Alena Akhmetshina,[1] Anita Pirchheim,[1] Margret Paar,[7] Beate Rinner,[8] Gerd Hörl,[7] Ernst Steyrer,[1] Ulrich Stelzl,[4,5] Boudewijn Burgering,[2] Tobias Eisenberg,[4,6,9] Brigitte Pertschy,[4,6,9] Dagmar Kratky,[1,4] and Tobias Madl[1,4,10,*]

[1]Gottfried Schatz Research Center for Cell Signaling, Metabolism and Aging, Molecular Biology and Biochemistry, Medical University of Graz, Neue Stiftingtalstraße 6/6, 8010 Graz, Austria

[2]Oncode Institute and Department of Molecular Cancer Research, Center for Molecular Medicine, University Medical Center Utrecht, 3584 CX Utrecht, the Netherlands

[3]Clinical Institute of Medical and Chemical Laboratory Diagnostics, Medical University of Graz, 8010 Graz, Austria

[4]BioTechMed-Graz, 8010 Graz, Austria

[5]Institute of Pharmaceutical Sciences, University of Graz, 8010 Graz, Austria

[6]Institute of Molecular Biosciences, NAWI Graz, University of Graz, 8010 Graz, Austria

[7]Otto-Loewi Research Center, Physiological Chemistry, Medical University of Graz, 8010 Graz, Austria

[8]Division of Biomedical Research, Medical University of Graz, 8036 Graz, Austria

[9]Field of Excellence BioHealth – University of Graz, Graz, Austria

[10]Lead contact

*Correspondence: tobias.madl@medunigraz.at

https://doi.org/10.1016/j.crmeth.2021.100016

MOTIVATION Protein arginine methylation (ArgMet) is of high current and increasing interest because of its fundamental role in regulation of cellular processes, including transcription, RNA processing, signal transduction cascades, the DNA damage response, and liquid-liquid phase separation. However, because of the lack of methods for global analysis of ArgMet, the mechanistic link between ArgMet levels, dynamics, and (patho)physiology remains largely unknown. Here, we took advantage of the high sensitivity and robustness of nuclear magnetic resonance spectroscopy and developed and applied a general method for quantification of global protein ArgMet.

SUMMARY

Quantitative information about the levels and dynamics of post-translational modifications (PTMs) is critical for an understanding of cellular functions. Protein arginine methylation (ArgMet) is an important subclass of PTMs and is involved in a plethora of (patho)physiological processes. However, because of the lack of methods for global analysis of ArgMet, the link between ArgMet levels, dynamics, and (patho)physiology remains largely unknown. We utilized the high sensitivity and robustness of nuclear magnetic resonance (NMR) spectroscopy to develop a general method for the quantification of global protein ArgMet. Our NMR-based approach enables the detection of protein ArgMet in purified proteins, cells, organoids, and mouse tissues. We demonstrate that the process of ArgMet is a highly prevalent PTM and can be modulated by small-molecule inhibitors and metabolites and changes in cancer and during aging. Thus, our approach enables us to address a wide range of biological questions related to ArgMet in health and disease.

INTRODUCTION

Arginine methylation (ArgMet) is a prevalent post-translational modification (PTM) evolutionarily conserved from unicellular eukaryotes to humans. It regulates a plethora of fundamental biological processes, such as transcription, translation, RNA metabolism, signal transduction, DNA damage response, apoptosis, and liquid-liquid phase separation (LLPS) (Bachand, 2007; Bedford and Clarke, 2009; Bedford and Richard, 2005; Lee et al., 2005; Pahlich et al., 2006).

Three main types of methylated arginine residues are present in cells, including ω-N^G-monomethylarginine (MMA), ω-N^G,N^G-asymmetric dimethylarginine (ADMA), and ω-N^G,N'^G-symmetric dimethylarginine (SDMA). Formation of MMA, SDMA, and ADMA is catalyzed by a broad spectrum of protein arginine methyltransferases (PRMTs). The number of PRMTs varies from unicellular eukaryotes to humans, and yeast has at least one or two main PRMTs (HMT1/RMT1 and HSL7) and a family of nine PRMTs being present in mammals (Bachand, 2007; Bedford and Clarke, 2009). Depending on the type of methylated arginine

This is an open access article under the CC BY license (http://creativecommons.org/licenses/by/4.0/).

they produce, PRMTs are categorized into four main classes (Bachand, 2007; Guccione and Richard, 2019). Type I PRMTs, including PRMTs 1, 2, 3, 4 (also called CARM1), 6, and 8, catalyze the formation of MMA/ADMA, whereas type II PRMTs, including PRMTs 5 and 9, catalyze the formation of MMA/SDMA (Figure S1A). Type III PRMTs such as PRMT7 catalyze the formation of MMA. In yeast, only the type IV PRMT RMT2 has so far been reported (Chern et al., 2002) to methylate the delta (δ) nitrogen atom of arginine residues (Niewmierzycka and Clarke, 1999). Additional potential arginine methyltransferases (NDUFAF7 and METTL23) have been identified, but remain to be biochemically validated (Guccione and Richard, 2019).

Most PRMTs methylate glycine- and arginine-rich, so-called arginine-glycine-glycine (RG/RGG), protein regions (Feng et al., 2013; Guo et al., 2014; Hamey et al., 2021). More than 1,000 human (in particular RNA-binding) proteins contain RG/RGG regions (Chong et al., 2018; Thandapani et al., 2013). However, adjacent glycine residues are not a prerequisite for the ArgMet as it has been shown that RXG motifs can be methylated by PRMT1, where X is preferably lysine, phenylalanine, threonine, or leucine (Uhlmann et al., 2012; Wooderchak et al., 2008). Moreover, RPAAPR or APR motifs have been identified as sites of ArgMet (Lee and Bedford, 2002). PRMT4/CARM1 has been reported to methylate arginines within proline-, glycine-, and methionine-rich regions (Cheng et al., 2007; Shishkova et al., 2017). A set of PRMT5 targets identified by mass spectrometry revealed the enzyme's preference for methylating arginine located between two neighboring glycines (GRG) (Musiani et al., 2019). PRMT6 prefers arginines in positively charged regions and disfavors acidic residues at essentially any position around the target arginines (Hamey et al., 2021). Within proteins, intrinsically disordered regions regularly display ArgMet but are not exclusive sites (Lorton and Shechter, 2019). On a molecular level, methylation of these regions regulates nucleic acid binding, protein-protein interactions, LLPS, and protein localization (Guccione and Richard, 2019).

PRMTs are ubiquitously expressed in human tissues (Scorilas et al., 2000), with the exception of PRMT8, mainly expressed in the brain (Lee et al., 2005), and regulate important cellular processes that affect cell growth, proliferation, and differentiation (Blanc and Richard, 2017). Embryonic loss of most of these PRMTs results in pre- and perinatal lethality in mice (Pawlak et al., 2000; Tee et al., 2010). Dysregulation of PRMTs has been implicated in the pathogenesis of several diseases, including cardiovascular, metabolic, and neurodegenerative diseases, viral infections, and various types of cancer (Blanc and Richard, 2017). Given that PRMTs tend to be upregulated in cancer malignancies (Jarrold and Davies, 2019; Yang and Bedford, 2013), they represent a promising target in cancer therapy and are currently being investigated in several clinical studies with PRMT inhibitors. Moreover, loss of PRMTs has been linked to cellular senescence and aging in mice (Blanc and Richard, 2017).

Despite the biological significance of ArgMet, several key questions are still elusive. (1) The global levels of ArgMet are largely unknown. Pioneering studies indicated that ArgMet might be as abundant as phosphorylation, and around 0.5%–2% of arginine residues are methylated in mammalian cells and tissues (Boffa et al., 1977; Esse et al., 2014; Matsuoka, 1972; Paik et al., 2007). Although more than 1,000 ArgMet sites have already been identified by immunoaffinity purification and liquid chromatography coupled with tandem mass spectrometry (Bremang et al., 2013; Guo et al., 2014), specific concentrations of ArgMet in cells and tissues, including the coupling of ArgMet and metabolism, have so far not been comprehensively studied by nuclear magnetic resonance (NMR). The methyl group for protein ArgMet is provided by the universal methyl donor *S*-adenosyl methionine (SAM), which is synthesized from methionine and ATP by SAM synthase. One-carbon metabolism is required for recycling of the essential amino acid methionine (Locasale, 2013; Yang and Vousden, 2016). How metabolism regulates ArgMet needs to be determined. (2) Dynamics and turnover of ArgMet, including the existence of an efficient arginine demethylase, are controversial and still largely unexplored issues (Guccione and Richard, 2019). (3) Regulators of PRMTs (e.g., BTG1, TIS21/BTG2, and NR4A1) were proposed in the last years, but their impact on PRMT activity and, in turn, their contribution to global ArgMet concentrations remains enigmatic (Bedford and Richard, 2005; Yang and Bedford, 2013). (4) Small-molecule inhibitors of PRMTs have been discovered, yet their influence on the extent of ArgMet and how ArgMet levels are affected *in vivo* is currently unknown.

Addressing these questions is challenging, in part due to the lack of robust methods for (absolute) quantification of global ArgMet values and dynamics in cells and tissues. Most of the current approaches use antibodies to detect and distinguish differentially methylated arginines. These methods successfully track and annotate these PTMs (Larsen et al., 2016). However, these antibodies are still only raised against specific, short target sequences (e.g., RGG) and mixtures of selected motifs, but fail to recognize or enrich the entire pool of arginine methylated proteins. This limits their use in quantifying of global ArgMet levels because of the large sequence diversities found around these sites (Bhatter et al., 2019; Lee and Stallcup, 2009).

We therefore developed a general method for absolute, label-free quantification of (methylated) arginines in cells, organoids, and tissues by using the high sensitivity and robustness of NMR spectroscopy. We demonstrate that ArgMet is a highly abundant PTM, whereas cellular dynamic changes of protein ArgMet occur at a slow rate. Our study provides a strong methodological development for the quantification of ArgMet levels and their dynamic changes that also conceptually advances our understanding of the importance of ArgMet in biology and medicine. Moreover, we offer ways to study the modulation of protein ArgMet by inhibitors, metabolites, and biological processes such as differentiation and aging, enabling future studies from basic to translational research and drug discovery/development far beyond the current state of the art.

RESULTS

NMR enables quantification of global protein arginine methylation

NMR spectroscopy enables robust quantification of metabolites in complex mixtures paired with simple and fast sample preparation, measurement, and analysis (Stryeck et al., 2018).

CellPress
OPEN ACCESS

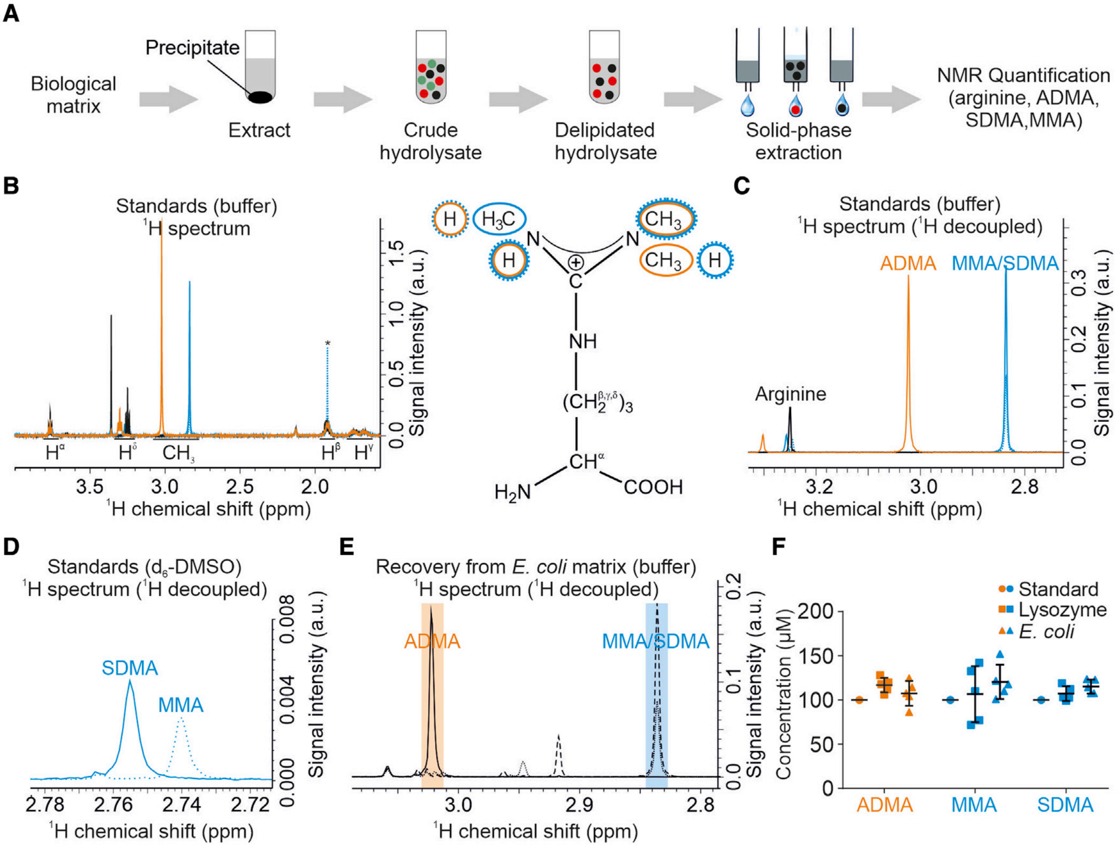

Figure 1. Absolute quantification of protein ArgMet by NMR

(A) Schematic workflow depicting steps for protein arginine and ArgMet quantification. Biological matrices are extracted with water/methanol. Protein precipitate containing protein arginine and ArgMet is hydrolyzed, lipids are removed with chloroform, and solid-phase extraction is used to isolate positively charged amino acids, including (methylated) arginine(s). The eluate is analyzed by NMR spectroscopy.

(B) Overlay of ^{1}H 1D-CPMG NMR spectra of 100 μM arginine (black), ADMA (orange), MMA (blue, dashed line) and SDMA (blue, solid line). Chemical-shift ranges for characteristic ^{1}H signals are shown in the spectra. Positions of the corresponding protons are labeled in the structure formula (ADMA, orange; MMA, blue dashed line, SDMA, blue solid line; an acetate impurity is labeled with an asterisk).

(C) Overlay of ^{1}H 1D projections of 2D J-resolved, virtually decoupled NMR spectra of the samples shown in (B). Characteristic regions of ADMA, MMA, and SDMA methyl groups are indicated (arginine, black; ADMA, orange; MMA, blue dashed line; SDMA, blue solid line).

(D) Overlay of ^{1}H 1D projections of 2D J-resolved NMR spectra of 100 μM MMA (blue dashed line) and SDMA (blue solid line) recorded in d_6-DMSO show the resolution of methyl resonances.

(E) Overlay of representative recovery experiments of ^{1}H 1D projections of 2D J-resolved NMR spectra recovery experiments from *E. coli* lysates spiked with ADMA (solid line), MMA (dashed line), or SDMA (dotted line), respectively. Shaded regions represent characteristic regions of ADMA (orange), and MMA and SDMA (blue) methyl groups.

(F) Statistical analysis of ADMA, MMA, and SDMA recovery from lysozyme (squares, n = 5; mean ± SD) (0.34 mM) and *E. coli* lysates (triangles, n = 5, mean ± SD). Samples were spiked with 100 μM ADMA (orange), MMA (blue), and SDMA (blue) and prepared according to the workflow shown in (A).

We built on previous chromatography-based approaches to analyze (methylated) arginines in protein hydrolysates (Dhar et al., 2013; Paik and Kim, 1967) and developed an NMR-based protocol for absolute quantification of protein ArgMet. A schematic representation of the workflow is shown in Figure 1A. Proteins were extracted from biological matrices, hydrolyzed by using hydrochloric acid, and delipidated. Basic/hydrophobic amino acids, including arginine and its derivatives, were purified by solid-phase extraction (SPE) and analyzed by NMR spectroscopy. NMR analysis of arginine, ADMA, MMA, and SDMA standards revealed good separation of their ^{1}H signals, in both one-dimensional (1D) Car-Purcell-Meiboom-Grill (CPMG) and two-dimensional (2D) homonuclear J-resolved experiments (JRES) (Figures 1B, 1C, and S1B). The JRES approach separates the chemical shift and J-couplings into two different spectral dimensions. To minimize signal overlap with other metabolites present in biological materials, we used the ^{1}H 1D projections of 2D J-resolved, virtually decoupled NMR spectra for all follow-up analyses, facilitating assignments and quantifications (Nagayama et al., 1977; Stryeck et al., 2018; Viant et al., 2003; Wang et al., 2003). ^{1}H-Methyl signals of MMA and SDMA overlapped in ^{1}H spectra when recorded in buffer, but could be resolved in deuterated dimethyl sulfoxide (d_6-DMSO) as solvent (Figure 1D).

To validate the robustness of our workflow, we first evaluated stability and recovery of ADMA, MMA, and SDMA signals in diverse biological matrices. All compounds were highly stable during hydrolysis and showed high recovery from both a protein matrix containing lysozyme and a methylation-free *Escherichia coli* cell matrix (Figures 1E, 1F, and S1E–S1G). Protein-unbound free methyl arginines did not contribute to the detected protein ArgMet (Figures S1C and S1D). A quantitation limit for ADMA of 100 nM was determined (Figure S1H). Concentrations remained linear over a wide concentration range of four orders of magnitude up to the SPE column saturation limit of 3 mM, as shown for arginine (Figure S1I). In summary, our NMR approach offers a simple, rapid, and highly reproducible workflow for arginine and ArgMet quantification. Compared with high-performance liquid chromatography (HPLC)-based quantification, NMR is label-free and does not require chromatographic separation or standards for quantification. Moreover, it enables detection of yet unknown arginine derivatives and can be combined with isotope labeling.

NMR-based protein ArgMet profiling *in vitro* and in cells

To identify the proportion of ArgMet in protein and cell samples of unknown methylation status, we determined levels of arginine, ADMA, MMA, and SDMA in recombinant proteins, yeast cell cultures, and mammalian cell lines. Levels of ADMA, MMA, and SDMA are presented as normalized to the total arginine content to allow a direct comparison of ArgMet concentrations between different biological matrices. Alternatively, and because NMR is completely quantitative, absolute concentrations can be displayed as normalized to either cell number, tissue mass, or protein content.

Methylation by PRMTs occurs preferentially within RG/RGG-rich and proline-glycine-methionine-rich regions (Blanc and Richard, 2017). In mammals, PRMT1 is the most abundant methyl transferase and catalyzes formation of both ADMA and MMA. As expected, NMR analysis of the methylation-free recombinant RG/RGG model proteins cold-inducible RNA-binding protein (CIRBP) and RNA-binding protein fused in sarcoma (FUS) revealed that ADMA and MMA are detectable in recombinant proteins after incubation with PRMT1 and the methyl donor SAM (Figure 2A). Both model proteins are suitable as *in vitro* substrates for PRMT1, and 12% and 5% of all arginine residues are asymmetrically dimethylated in CIRBP and FUS, respectively. Interestingly, the levels of ADMA and MMA varied between CIRBP and FUS, with CIRBP lacking MMA and FUS showing MMA (Figure S2A). The increased content of MMA in FUS might be due to the presence of two RGGY motifs in FUS. A preference for tyrosine in the +3 position was observed for PRMT1 MMA target sites (Hartel et al., 2019). We cannot rule out the possibility that *in vitro* methylation might lead to high MMA levels. These data indicated that PRMT1 selectively recognizes amino acid sequences in substrate (Guo et al., 2014) and that ArgMet NMR is well applicable to study levels and kinetics of ArgMet in purified protein substrates.

First HPLC-based studies estimated 0.5%–2% of arginine residues to be methylated in mammalian cells and tissues (Boffa et al., 1977; Esse et al., 2014; Matsuoka, 1972; Paik et al., 2007). In yeast, four PRMTs (HMT1/RMT1 [Gary et al., 1996; Henry and Silver, 1996], RMT2 [Niewmierzycka and Clarke, 1999], HSL7 [Miranda et al., 2006], and SFM1[Young et al., 2012]) have been described. Additionally, a large number of methylation sites and their associated proteins have been identified by mass spectrometry (Erce et al., 2013; Plank et al., 2015), suggesting that ArgMet might represent an important mechanism in yeast. Of these PRMTs, HMT1/RMT1 has already been identified as a PRMT1 homolog in 1996 (Gary et al., 1996; Henry and Silver, 1996). Analysis of wild-type and *HMT1* or *HSL7* knockout yeast strains, assessed in two distinct but related genetic backgrounds (BY4741 and BY4742), showed that on average more than 0.25% of all arginines are methylated in *S. cerevisiae* (Figure 2B). MMA was detectable in wild-type yeast (BY4741 and BY4742), albeit at low levels (~20% of ADMA), whereas SDMA was undetectable (Figures 2C and S2B). Deletion of *HMT1* essentially abolished ADMA and MMA levels in both backgrounds, consistent with an HPLC-based validation experiment (Figure S2B), suggesting that none of the other PRMTs contributed significantly to the global ArgMet levels. In line with these results, only very few substrates of RMT2, HSL7, and SFM1 have been reported so far (Chern et al., 2002; Sayegh and Clarke, 2008; Young et al., 2012). In contrast to *HMT1* deletion, deletion of *HSL7* showed no significant impact on global ADMA and MMA levels in BY4741 and BY4742 (Figure 2B), probably because HSL7 only recognizes a small subset of potential substrate proteins in yeast (Sayegh and Clarke, 2008). Indirect effects associated with the loss of *HMT1* are unlikely, as the expression of other PRMTs is not affected by *HMT1* knockout (Chia et al., 2018)

Our approach offers an excellent opportunity to characterize ArgMet in a variety of commonly used human cell lines. ArgMet-NMR analysis of nine human cells lines showed that ADMA and MMA/SDMA concentrations differ significantly; primary fibroblasts showed the lowest, and A375 malignant melanoma cells showed the highest levels of both ADMA and MMA/SDMA, respectively (Figure 2D). In all cell lines tested, ADMA was the predominant ArgMet species with more than 3% of all arginine residues being methylated in A375 cells. SDMA/MMA levels were significantly lower (Figures 2D and 2E). This finding is in line with previous studies estimating MMA and SDMA at levels of 20%–50% of ADMA (Bedford and Clarke, 2009), although the MMA/SDMA values detected by NMR are consistently lower (~10% of ADMA). The significantly increased concentrations of ArgMet in A375 cells compared with all other cell lines is in agreement with a recent study showing overexpression of PRMT1 in these cells (Li et al., 2016). In contrast, HPLC-based methods detected 0.8% of all arginine residues in A375 cells being asymmetrically dimethylated, which is lower than the 3.4% of ADMA we found (Bulau et al., 2006). The increase of ADMA in all investigated cell lines is correlated with a concomitant increase in MMA and SDMA, indicating that the corresponding enzymes might be coregulated (Figure S2C). Nevertheless, we cannot exclude that the level of substrate proteins and PRMTs might also affect the ArgMet levels. PRMTs are constitutively active and localized in the nucleus and cytoplasm (Goulet et al., 2007; Herrmann et al., 2009). In the nucleus, histone ArgMet is an important modulator of dynamic chromatin regulation and transcriptional controls

CellPress
OPEN ACCESS

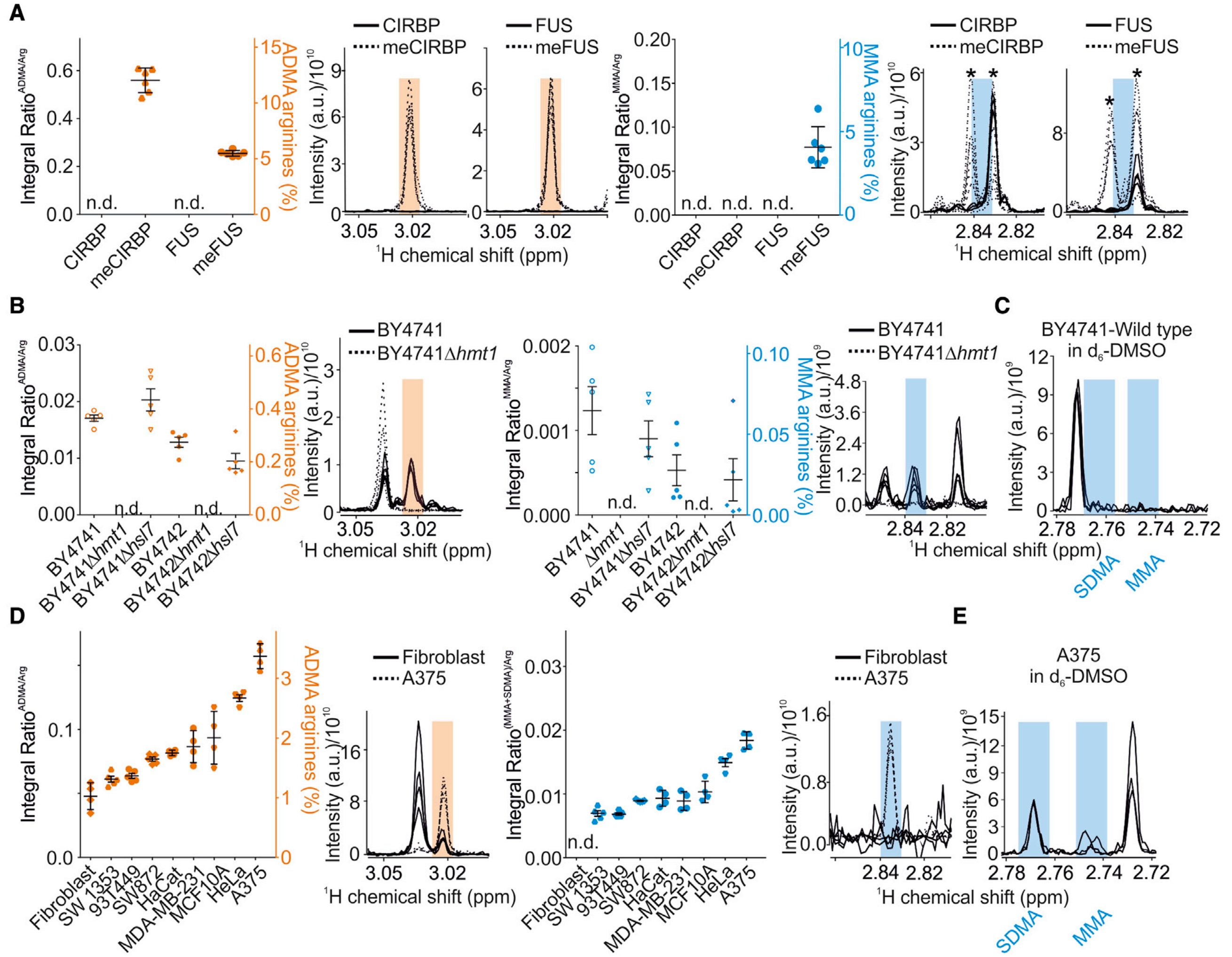

Figure 2. Characterization of ArgMet in purified proteins, yeast, and mammalian cell lysates

(A) ArgMet quantification of recombinant CIRBP (triangles) and FUS (circles) peptides without methylation or *in vitro* methylated by recombinant PRMT1, respectively (n = 6; mean ± SD; n.d., not detectable; Tris buffer impurities are labeled with asterisks).

(B) Protein ArgMet quantification of yeast lysates obtained from logarithmically grown wild-type (BY4741 and BY4742) and respective *HMT1* (Δ*hmt1*) or *HSL7* (Δ*hsl7*) knockout cells (n = 5; mean ± SD; n.d., not detectable).

(C) Spectral overlays of characteristic MMA and SDMA NMR methyl signals in d_6-DMSO show that MMA and SDMA methyl resonances can be resolved (n = 3).

(D) Protein ArgMet quantification of human cell lysates (n = 4–5; mean ± SD; n.d., not detectable). ADMA levels in relation to the total amount of arginine are indicated. Spectral overlays of characteristic ADMA and MMA/SDMA NMR methyl signals are shown (n = 4).

(E) Spectral overlays of characteristic MMA and SDMA NMR methyl signals in d_6-DMSO show that MMA and SDMA methyl resonances can be resolved (n = 3).

In (A), (B), and (D), the ADMA concentrations are indicated in relation to the total amount of arginine. Spectral overlays of characteristic ADMA (orange) and MMA/SDMA (blue) NMR methyl signals are shown as shaded regions (n = 4–6).

(Litt et al., 2009). We therefore analyzed the ArgMet levels of chromatin and cytoplasm in A375 and HeLa cells and observed a significant increase of ArgMet in the chromatin fractions compared with the cytoplasmic fractions or whole-cell lysates (Figures S2D and S2E). This is in agreement with a previous study identifying lower PRMT1 protein levels in the chromatin fractions compared with the cytoplasm in HeLa cells (Musiani et al., 2020). The higher levels of ArgMet observed in chromatin might be because of a higher proportion of well-established PRMT substrates, such as histone proteins, which can be methylated by multiple PRMTs (Bedford, 2007). Our observation that ArgMet levels in the cytoplasm are similar to ArgMet levels in whole cells confirmed that the high ArgMet content in whole cells is not because of the chromatin compartment but to an overall high ArgMet level.

Generally, non-cancer cell lines such as primary fibroblasts show a tendency to lower concentrations of ArgMet compared with cancer cell lines such as HeLa, A375, or MDA-MB-231. Although HaCaT cells, an immortalized human keratinocyte line, also exhibit higher ArgMet levels, the values are still lower than in cancer cell lines. In summary, our approach provides a direct readout of protein ArgMet in cell lines.

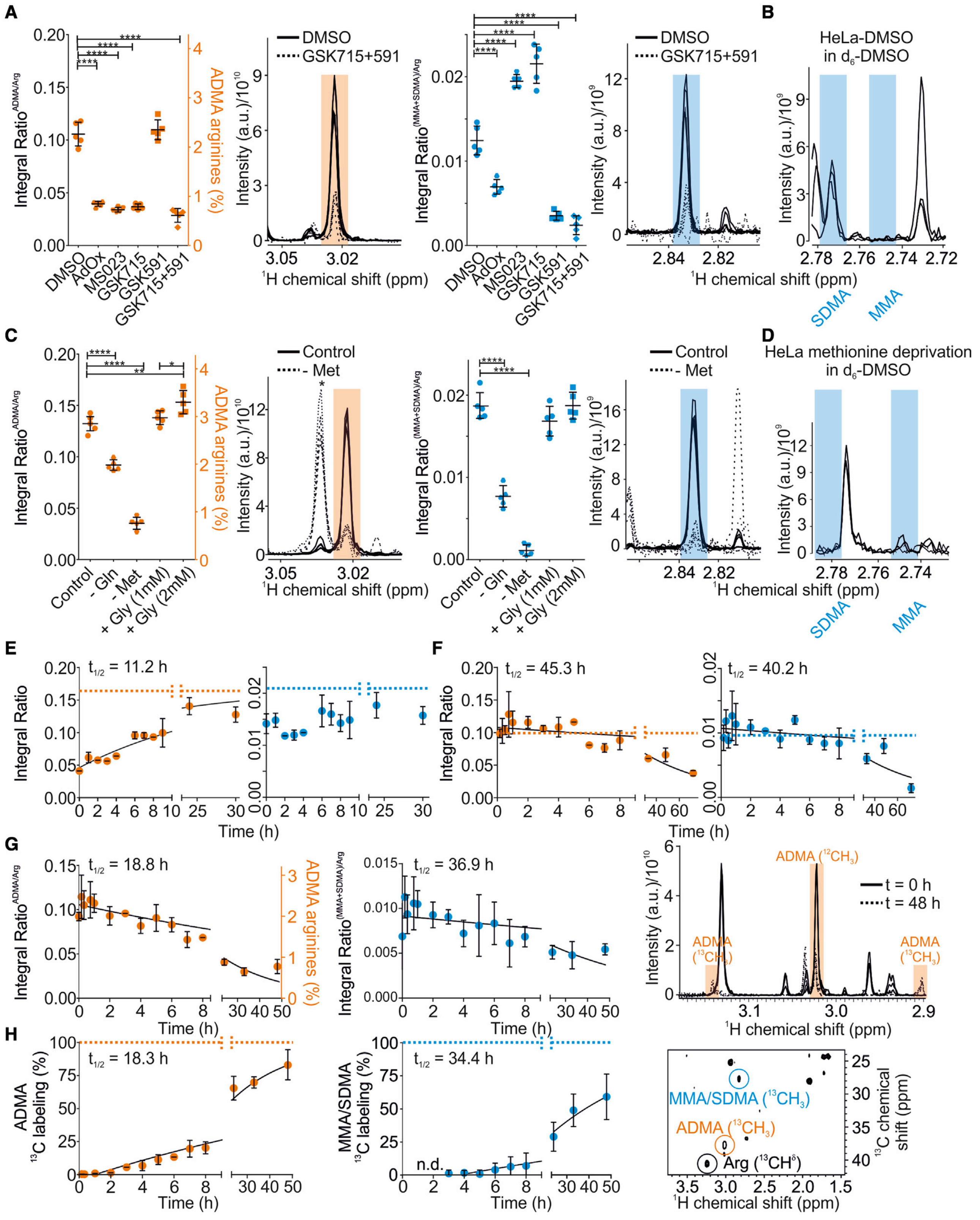

(legend on next page)

CellPress
OPEN ACCESS

NMR reveals modulation and dynamics of protein ArgMet

Although it has taken 50 years to acknowledge the significance of PRMTs in cancer, the pace at which major discoveries have been made in recent years is phenomenal. Disruption of ADMA modification at key substrates decreases the metastatic and proliferative ability of cancer cells (Li et al., 2016), suggesting that PRMT inhibitors might be an effective strategy to combat different types of cancer. Several PRMT inhibitors have entered or are on the verge of entering the clinic, but how they alter global protein ArgMet levels remains to be uncovered.

Given that our method provides a direct readout of ArgMet modulation by PRMT inhibitors, we characterized ArgMet concentrations under distinct conditions of PRMT inhibition (Figures 3A and 3B). We first tested the impact of the commonly used general ArgMet inhibitor adenosine dialdehyde (AdOx) and the type I PRMT inhibitor MS023 (Afman et al., 2005; Chan-Penebre et al., 2015; Guccione and Richard, 2019). In line with our hypothesis, AdOx unselectively, though incompletely, reduced any kind of protein ArgMet significantly by ~60% ($p < 0.0001$). As expected for a selective type I PRMT inhibitor, MS023 inhibited mostly ADMA, but not SDMA formation. PRMT5, the major enzyme catalyzing the formation of SDMA, has been implicated in cancer biology, and controls expression of both tumor-suppressive and tumor-promoting genes (Guccione and Richard, 2019). Inhibition of PRMT5 by the small-molecule compounds GSK3203591 or GSK3326595 has been reported to act antiproliferatively on mantle cell lymphoma, both *in vivo* and *in vitro* (Chan-Penebre et al., 2015; Gerhart et al., 2018). Moreover, GSK3368715, a reversible type I PRMT inhibitor, exhibited antitumor effects in human cancer models and is currently in phase I clinical trials (Guccione and Richard, 2019). GSK3203591 and GSK3368715 have been reported to synergistically inhibit tumor growth *in vivo*, possibly through a tumor-specific accumulation of 2-methylthioadenosine, an endogenous inhibitor of PRMT5, which correlates with sensitivity to GSK3368715 in cell lines (Fedoriw et al., 2019). In agreement, GSK3368715 inhibited formation of ADMA but not SDMA formation (Figure S3A), whereas GSK3203591 inhibited generation of MMA/SDMA but not of ADMA. These results were further validated by reverse HPLC (Figure S3B). Compared with all other conditions tested, a combination of GSK3203591 and GSK3368715 showed the strongest inhibition of any type of ArgMet in HeLa cells. Interestingly, inhibition of type I PRMTs by MS023 or GSK3368715 doubled the levels of SDMA/MMA, suggesting that, on a global scale, several type I PRMT targets become symmetrically instead of asymmetrically dimethylated. Accordingly, recent western blotting experiments resulted in increased MMA/SDMA levels after treatment with PRMT1 inhibitors (Dhar et al., 2013; Eram et al., 2016; Fedoriw et al., 2019). Comparable results in other cell lines demonstrated that the mechanisms of ArgMet inhibition are independent of the cell line (Figures S3C and S3D).

One of the main goals of current ArgMet research is to further refine our mechanistic understanding of ArgMet and how this process is coupled with metabolism. PRMTs add methyl groups to arginine residues by using the universal methyl donor SAM, which is recycled through one-carbon metabolism (Ducker and Rabinowitz, 2017; Yang and Vousden, 2016). Methionine is a key substrate for SAM production. Beyond the single-carbon metabolic pathway, additional metabolites can alter global cellular ArgMet by modulating the SAM levels and recycling.

Figure 3. NMR enables quantification of protein ArgMet modulation and dynamics

(A) Protein ArgMet quantification of HeLa cells treated for 3 days with either DMSO, 40 μM adenosine dialdehyde (AdOx), 10 μM MS023, 2 μM GSK3368715 (GSK715), 1 μM GSK3203591 (GSK591), or a combination of 2 μM GSK715 and 1 μM GSK591 (n = 5; mean ± SD; *$p < 0.05$, **$p < 0.01$, ***$p < 0.001$, ****$p < 0.0001$). ADMA levels in relation to the total amount of arginine are indicated. Spectral overlays of characteristic ADMA (orange) and MMA/SDMA (blue) NMR methyl signals are shown as shaded regions (n = 5).

(B) Spectral overlays of characteristic MMA and SDMA NMR methyl signals in d_6-DMSO show that MMA and SDMA methyl resonances can be resolved (n = 3). Shaded regions represent characteristic regions of MMA and SDMA (blue) methyl groups.

(C) Protein ArgMet quantification of HeLa cells cultured with or without 4 mM glutamine (Gln), 0.2 mM methionine (Met), or glycine (1 mM and 2 mM Gly) (n = 5; mean ± SD; *$p < 0.05$, **$p < 0.01$, ***$p < 0.001$, ****$p < 0.0001$). ADMA levels in relation to the total amount of arginine are shown. Spectral overlays of characteristic ADMA (orange) and MMA/SDMA (blue) NMR methyl signals are presented as shaded regions (n = 5). Unmethylated lysines are labeled with asterisks.

(D) Spectral overlays of characteristic MMA and SDMA NMR methyl signals in d_6-DMSO show that SDMA levels strongly decrease upon methionine deprivation (n = 3).

(E) Changes of ArgMet levels after removal of AdOx. Prior to removal of AdOx, HeLa cells were treated with AdOx for 3 days to reduce ArgMet. Integral ratios of ADMA/arginine (orange) and (SDMA + MMA)/arginine (blue) are plotted as mean ± standard error (n = 3) for each time point. To estimate the half-life of ArgMet recovery ($t_{1/2}$), the data were fitted by using a single exponential recovery function (95% confidence interval [CI] 7.5–19.9 h). Dotted lines indicate the level of methylation in the absence of AdOx.

(F) Changes of ArgMet levels after methionine removal. Integral ratios of ADMA/arginine (orange) and (SDMA + MMA)/arginine (blue) are plotted as mean ± standard error (n = 3) for each time point. To estimate the half-life of ArgMet decay ($t_{1/2}$), the data were fitted by using a single exponential decay function (ADMA/arginine: 95% CI 34.0–62.7 h; (SDMA + MMA)/arginine: 95% CI 27.8–61.1 h). Dotted lines indicate the level of methylation in the presence of methionine.

(G) Dynamics of *de novo* ArgMet via ^{13}C labeling are shown as decay of the ^{12}C-methyl NMR signals upon exchange of media containing ^{13}C-methyl-labeled methionine. Integral ratios of ADMA/arginine (orange) and (SDMA + MMA)/arginine (blue) are plotted as mean ± standard error (n = 3) for each time point. To estimate the half-life of ArgMet ^{12}C-methyl signal decay ($t_{1/2}$), the data were fitted by using a single exponential decay function (ADMA/arginine, 95% CI 14.4–24.7; (SDMA + MMA)/arginine, 95% CI 21.9–73.8). Change of ADMA $^{12}C/^{13}C$-methyl signals at the beginning and after 48 h of cultivation in presence of ^{13}C-methyl-labeled methionine (^{1}H 1D projections of 2D J-resolved NMR spectra).

(H) Dynamics of *de novo* ArgMet via ^{13}C labeling are shown as increase of the ^{13}C-methyl NMR signals detected in $^{1}H,^{13}C$ HSQC (heteronuclear single quantum coherence spectroscopy) NMR spectra upon exchange of media containing ^{13}C-methyl-labeled methionine. Fractions of ^{13}C labeling are plotted as mean ± standard error (n = 3) for each time point. To estimate the half-life of ArgMet ^{13}C-methyl labeling ($t_{1/2}$), the data were fitted by using a one-phase association function (ADMA/arginine, 95% CI 16.4–21.2; (SDMA + MMA)/arginine, 95% CI 27.4–43.5). Arginine (black), ADMA (orange), MMA, and SDMA (blue) $^{1}H,^{13}C$ NMR signals are labeled in a representative $^{1}H,^{13}C$ HSQC NMR spectrum.

Indeed, considering that methionine is an essential amino acid and its recycling can therefore only partly contribute to the methionine pool required for SAM generation, deprivation of methionine strongly reduced the concentrations of ADMA and MMA/SDMA in HeLa cells (Figures 3C and 3D). Production of SAM requires ATP, and its recycling via *S*-adenosyl-homocysteine depends on supply of the single-carbon building block from serine (Yang and Vousden, 2016). In cancer cells, glutamine can provide both the single-carbon building block through gluconeogenesis and energy through the tricarboxylic acid cycle. Thus, we tested in HeLa cells if depletion of glutamine reduced the overall levels of protein ArgMet. In line with our hypothesis, concentrations of ADMA and SDMA/MMA were reduced, although not as profoundly as in the case of methionine withdrawal. Glycine supplementation has been proposed to mimic the effects of methionine deprivation through inhibition of the serine-to-glycine conversion that otherwise provides the single-carbon building block for SAM recycling (Partridge et al., 2020). In contrast to these studies, we even observed an increase in ADMA when cells were incubated with 2 mM glycine (Figure 3C). Taken together, ArgMet NMR provides a toolbox for future studies of protein ArgMet regulation by inhibitors and metabolites.

Dynamics of arginine methylation and demethylation is one of the yet unsolved and controversial questions in the field (Guccione and Richard, 2019). Several enzymes have been reported to act as demethylases. Peptidylarginine deiminase 4 (PAD4) might "demethylate" proteins by converting methylated arginine to citrulline (Wang et al., 2004). The Jumonji domain-containing 6 (JMJD6) protein has been reported to demethylate arginine in histone tails (Chang et al., 2007). Nevertheless, both demethylation pathways remain controversial.

We therefore addressed the dynamics of remethylation in a low-ArgMet background. We treated cells with medium supplemented with AdOx to reduce ArgMet, then changed the medium to AdOx-free medium and collected cells at different time points. We found that levels of ArgMet recovered slowly after AdOx removal, and ADMA had a half-life of >11 h (Figure 3E). As this process might have been affected by the levels of AdOx decreasing slowly inside the cell, we further validated the changes in ArgMet concentrations by using methionine deprivation. Under these conditions, levels of protein ArgMet decreased considerably (~60%), with half-lives of 45 h and 40 h for ADMA and MMA/SDMA, respectively (Figure 3F). Although these alterations are strongly coupled to the dynamics of the cellular pool of methionine, our results indicate that demethylation of methylated arginine residues is a slow process and that the available levels of methionine are insufficient to maintain the methylation levels over a longer period of time.

To monitor the dynamics of ArgMet in the absence of any interference due to the manipulation of metabolic pathways, we combined ArgMet NMR with stable isotope tracing by using ^{13}C-methyl-labeled methionine. With methionine being an essential substrate for SAM production, we next examined whether the methyl group crucial for ArgMet is donated by methionine and investigated the dynamics of the associated methylation reaction. To track and quantify *de novo* ArgMet, we pulsed HeLa cells in media with ^{13}C-methyl-labeled methionine and chased its appearance by the decay of the ^{12}C-methyl NMR signals upon exchange with media containing ^{13}C-methyl-labeled methionine (Figures 3G and 3H). Coupled with the decrease of ^{12}C protein ArgMet, "newly" synthesized and ^{13}C isotopically labeled protein ArgMet appears (Figure 3H). Fitted half-lives of demethylation (^{12}C-decay) and *de novo* methylation (^{13}C-increase) were in excellent agreement and approximately 18–19 h and 34–37 h for ADMA and MMA/SDMA, respectively. In line with the AdOx removal and methionine deprivation changes, these data indicate that the overall dynamics of arginine demethylation are slow.

NMR provides insights into dynamics of ArgMet in organoids and tissues

Increasing evidence suggests that ArgMet is required to maintain cells in a proliferative state and plays a key role in the homeostasis of stem cell pools (Blanc and Richard, 2017). In addition, the role of PRMTs has been associated with cell growth, differentiation, apoptosis, and aging (Blanc and Richard, 2017; Guccione and Richard, 2019; Yang and Bedford, 2013). For example, depletion and exhaustion of muscle and hematopoietic stem cells in adulthood was linked to loss of ArgMet (Blanc et al., 2016; Liu et al., 2015). In addition, PRMTs play important regulatory roles in the differentiation of myeloid cells (Balint et al., 2005). To study the relationship of ArgMet and *in vitro* differentiation in a controlled manner, we generated cell-type-enriched mouse small intestinal organoid cultures. We grew the organoids in complete ENR medium (EGF, Noggin, R-Spondin) as reference. In ENR medium, organoids contain stem cells, enterocytes, and Paneth cells (roughly 15%, 80%, and 5%, respectively). Stem cells, enterocytes, and Paneth cells were enriched by using media supplemented with Wnt-CM (conditioned medium)/valproic acid (VPA; stem cells enriched), removal of R-Spondin (EN; enterocytes enriched), or supplementation of Wnt-CM/*N*-[*N*-(3,5-difluorophenacetyl)-L-alanyl*]-*S*-phenylglycine t-butyl ester) (DAPT; Paneth cells enriched), respectively. Our data show alterations of ADMA and MMA/SDMA dependent on organoid composition (Figure 4A). In line with a high expression of PRMTs in stem cells found in single-cell mRNA sequencing of mouse small intestine (Haber et al., 2017; Ludikhuize et al., 2020; Uhlen et al., 2015) (Figures S4A–S4C, http://www.proteinatlas.org/), reference organoids (ENR) show higher ADMA and MMA/SDMA. Enrichment of Paneth cells in organoids (DAPT) results in a strong decrease in overall ArgMet, in line with a low expression of PRMTs in Paneth cells (Figures S4A–S4C). However, it remains to be investigated whether ArgMet is a cause or consequence of differentiation and to elucidate the key regulatory and metabolic mechanisms modulating ArgMet during differentiation.

Studying the global levels of ArgMet *in vivo* is the ultimate goal to reveal the mechanistic links between ArgMet and (patho) physiology. To demonstrate the feasibility of our approach for *in vivo* studies, we characterized ArgMet levels in commonly studied mouse tissues (brain, heart, kidney, liver, spleen, and lung) in two groups of female wild-type mice (mixed background of 129/J and C57BL/6J) at young (9–11 weeks) and old (96–104 weeks) age. Strikingly, we observed varying levels of ADMA and MMA/SDMA among tissues, and the highest levels

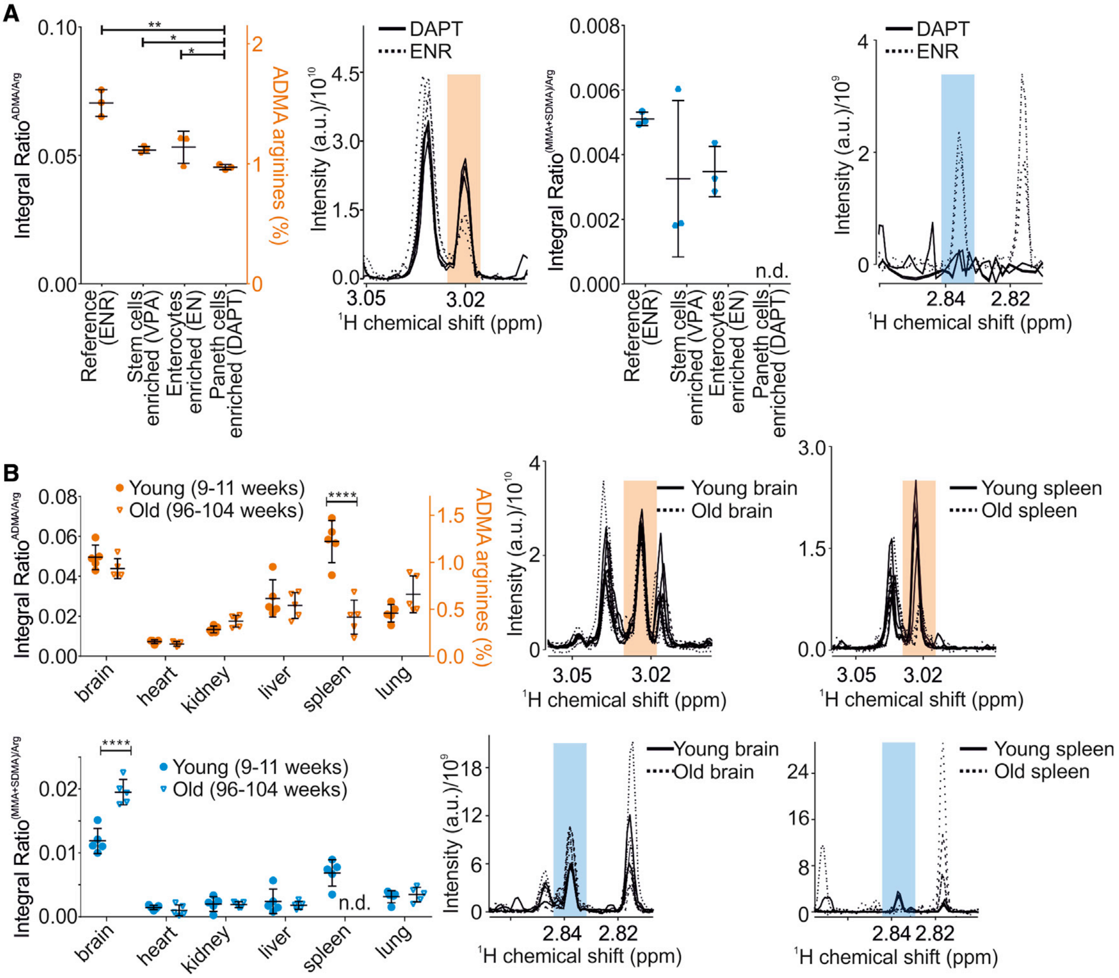

Figure 4. NMR enables characterization of ArgMet in cell differentiation and aging *in vivo*

(A) Protein ArgMet quantification of mouse small intestinal organoids cultured with EGF/Noggin/R-spondin1 (ENR), EGF/Noggin (EN), ENR plus valproic acid (VPA), or ENR plus Notch pathway inhibitor DAPT (n = 3; mean ± SD; *p < 0.05, **p < 0.01, ***p < 0.001, ****p < 0.0001). ADMA levels are presented with respect to total amounts of arginine. Spectral overlays of characteristic ADMA (orange) and MMA/SDMA (blue) NMR methyl signals are shown as shaded regions (n = 3).

(B) Quantification of protein ArgMet in mouse tissues collected from young mice (9–11 weeks, dots) and old mice (96–104 weeks, triangles) (n = 5; mean ± SD; *p < 0.05, **p < 0.01, ***p < 0.001, ****p < 0.0001). ADMA levels are presented with respect to total amounts of arginine. Spectral overlays of characteristic ADMA (orange) and MMA/SDMA (blue) NMR methyl signals are shown as shaded regions (n = 3).

of ArgMet were in brain and spleen of young mice (Figure 4B). Recent studies revealed high expression of PRMT1 in the rat spleen and high expression of PRMT5 in the rat brain (Hong et al., 2012), substantiating our findings of high ADMA in the spleen and high MMA/SDMA in the brain. Moreover, PRMT1 and PRMT8 expressions were elevated in mouse brain compared with liver (Wang et al., 2017). The PRMT7 mRNA expression in the spleen in old mice was markedly reduced (Figure S4H), consistent with the decreased ArgMet level in old mice. However, overall PRMT mRNA expression levels were not associated with protein ArgMet levels in the tissues tested (Figures S4D–S4I). With the exception of brain and spleen, we observed no significant aging-related changes in ArgMet levels in any other tissues. Expression and catalytic activity of PRMT1, PRMT4, PRMT5, and PRMT6 have been reported to be reduced in replicatively senescent cells in relation to young cells (Lim et al., 2008, 2010). In addition, senescent cells accumulate in tissues with age along with a decline in immune function (Kuilman et al., 2008). Although the underlying molecular mechanisms remain elusive, one might speculate that the changes in the aged spleen are caused by the accumulation of senescent cells.

DISCUSSION

Protein ArgMet modulates the physicochemical properties of proteins and thus plays a major role in a multitude of regulatory pathways, including gene regulation, signal transduction, regulation of apoptosis, and DNA repair (Blanc and Richard, 2017; Guccione and Richard, 2019; Yang and Bedford, 2013). Although previous studies exist in this field, the lack of a reliable quantification of ArgMet is a restricting factor in elucidating the relevance of ArgMet in physiological and pathological

processes. We have developed a simple, fast, and robust protocol for NMR-based quantification of protein ArgMet levels and dynamics in purified proteins, cells, organoids, and tissues. Our study reveals that NMR spectroscopy provides a sensitive readout for detection and quantification of MMA, ADMA, and SDMA in all matrices tested. We show that ArgMet NMR enables detection of methylation patterns in purified proteins incubated with PRMT1. Methylation by PRMTs in the human proteome occurs preferentially (but not exclusively) within glycine-arginine-rich and proline-glycine-methionine-rich regions (Cheng et al., 2007; Thandapani et al., 2013; Woodsmith et al., 2018), but specific consensus sequences targeted by most of the human PRMTs remain to be identified. Our approach provides a toolbox for fast and label-free screening for PRMT selectivity in purified proteins/peptides, complementary to peptide arrays (Kusevic et al., 2016) and mass spectrometry (Uhlmann et al., 2012).

Although some human PRMTs are well studied, for a plethora of PRMTs from other organisms it is as yet unknown whether they exhibit any enzymatic activity (Fulton et al., 2019). For example, the main yeast methyltransferase is HMT1, the presumable ortholog of human PRMT1. In addition, *in silico* studies have predicted 33 additional putative methyltransferases in *S. cerevisiae*, and it is likely that besides nucleic acid methyltransferases and protein methyltransferases specific to other amino acids, arginine methyltransferases are also among them (Low and Wilkins, 2012). We found that *S. cerevisiae* produces ADMA and MMA, but no SDMA, in line with a previous report (Hsieh et al., 2007). Strikingly, deletion of *HMT1* led to a complete loss of ADMA and MMA, suggesting that the contribution of any other methyltransferase to global levels of ArgMet is negligible, at least in these yeast strains. However, we cannot exclude the possibility that the other putative methyltransferases methylate only a small subset of targets, resulting in low global ArgMet levels. Our proof-of-principle analysis in human cell lines identified a large fraction of arginine residues in a methylated state, ranging from 1% to 3.4%. In all cell lines tested, ADMA constituted the predominant methylated arginine species, followed by SDMA with about 10% and MMA with about 1% of ADMA. These ADMA levels are in agreement with the findings that PRMT1 is the predominant and most active PRMT present in mammalian cells (Tang et al., 2000). Screening the PhosphoSitePlus database of PTMs for ArgMet revealed that for 1.7% of all arginines in human proteins, methylation (ADMA, SDMA, or MMA) has been reported. Given that we identified between a methylation status of 1% and 3.4% of arginines to be methylated indicates that most of the proteins for which ArgMet has been reported are entirely methylated. Note that this estimation assumes that all proteins are present at comparable levels inside the cell (Hornbeck et al., 2015). Methylarginines are predominantly found in intrinsically disordered protein regions, e.g., RG/RGG regions, which are intimately connected to LLPS (Chong et al., 2018; Guccione and Richard, 2019; Woodsmith et al., 2018). A large proportion of the proteins implicated in LLPS are known targets for ArgMet and, therefore, LLPS could be regulated by their ArgMet (Chong et al., 2018; Guccione and Richard, 2019; Lorton and Shechter, 2019). Thus, it is conceivable that the global ArgMet levels regulate LLPS on a global scale *in vivo* by regulating fluidity and dynamics of membrane-less organelles containing, for example, RG/RGG proteins.

By comparing the methylation levels in cell lines, ArgMet were up to 3-fold higher in immortalized and cancer cells compared with primary cells. Strikingly, cells isolated from human metastases contained the highest levels of protein ArgMet. The increased ArgMet levels found in cancer cells are in line with overexpression of PRMT1 in human melanoma, breast, and prostate cancer (Bedford, 2007; Hamamoto and Nakamura, 2016). In addition, PRMT5 expression and activity seem to be important in tumorigenesis and are markers of poor clinical outcome (Stopa et al., 2015). Based on the observation that increased PRMT expression is associated with tumor growth, inhibitors of protein arginine methyltransferases have been developed and showed promising results in clinical studies (https://clinicaltrials.gov). Our study demonstrates that ArgMet NMR provides a precise and specific readout for modulation of ArgMet levels in cells treated with distinct (specific) PRMT inhibitors. This suggests that ArgMet NMR might be a valuable tool for ArgMet-based drug discovery, drug validation, and patient stratification in the future.

By examining the modulation of ArgMet levels upon metabolite deprivation in cells, we detected a tight metabolic regulation of ArgMet levels by methionine, glutamine, and glycine. Methionine is required for protein synthesis and its adenylation produces SAM, which serves in turn as a methyl donor for methylation reactions (Locasale, 2013; Yang and Vousden, 2016). Accordingly, we demonstrated that methionine deprivation had a strong impact on protein ArgMet by reducing ADMA, MMA, and SDMA by more than 61%. Given that methionine is an essential amino acid whose levels are dictated by dietary factors (Mentch and Locasale, 2016), it is conceivable that nutrition and fasting could, in addition to protein synthesis, additionally affect protein ArgMet *in vivo*. Moreover, glutamine deprivation in HeLa cell culture reduced protein ArgMet by more than 30%, corroborating the observation that glutamine is a key energy source in cancer cells and can provide the single-carbon building block for SAM recycling through gluconeogenesis (Curi et al., 2005). Glutamine plays a pleiotropic role in cellular function and its consumption is elevated in proliferating cells not only because of increased DNA production (Counihan et al., 2018; Vander Heiden and DeBerardinis, 2017) but also for maintaining high ArgMet levels. Notably, ArgMet requires an energy demand of 12 molecules of ATP per methylation event (Gary and Clarke, 1998). Thus, reduced energy supply by glutamine deprivation could be the major factor in the observed reduction of protein ArgMet. Glycine is an interesting metabolite owing to its role in SAM recycling and methionine clearance. On the one hand, glycine can act as methyl group acceptor, leading to the formation of sarcosine (*N*-methylglycine) and *S*-adenosylhomocysteine. On the other hand, glycine is converted when the single-carbon block is transferred to tetrahydrofolate, which in turn is used to recycle SAM (Ducker and Rabinowitz, 2017; Luka et al., 2009; Yang and Vousden, 2016). Excess glycine has been proposed to reduce methionine levels and to mimic methionine deprivation (Partridge et al., 2020). According to our findings but in contrast to previous studies, glycine supplementation failed to reduce global levels of protein ArgMet. The fact that

glycine supplementation did not alter methionine levels in adult worms (Liu et al., 2019) suggests that under physiological conditions glycine supplementation is not generally applicable to mimic methionine deprivation in cancer cells. Our approach is expected to substantiate specific aspects of protein methylation research in the future. Moreover, it will be interesting to reveal whether lifespan extension via methionine restriction is mediated by modulation in ArgMet (Bárcena et al., 2018; Grandison et al., 2009; Lee et al., 2016).

Dynamics of cellular protein ArgMet, the process of methylation and the process of "demethylation," can also be easily examined by our methodology. The existence of an efficient arginine demethylase has not yet been proved and is a long-disputed question in this field (Low et al., 2016). Our results obtained by using different setups of remethylation after treatment with the general methylation inhibitor AdOx and "demethylation" upon methionine deprivation show that global arginine (de) methylation is a slowly developing process in a cellular context. We further substantiated these findings by combining ArgMet NMR with stable isotope tracing by using ^{13}C-methyl-labeled methionine. A decrease of the NMR signal characteristic for unlabeled methylated arginine residues in combination with an increase of the NMR signal characteristic for ^{13}C-methylated arginines indicated that both *de novo* ArgMet and "demethylation" are slow processes, especially in comparison with phosphorylation and dephosphorylation. For example, global phosphorylation of the epidermal growth factor receptor occurs within 2–3 h with a half-life of approximately 30 min, whereas its intracellular domain is dephosphorylated considerably faster ($t_{1/2}$ = 15 s) (Gelens and Saurin, 2018). We therefore conclude that no efficient demethylase exists that affects global methylation levels in HeLa cells. Whether demethylation affects specific targets rather than the global ArgMet levels remains to be investigated.

We observed even in mouse tissues a large fraction of arginines being methylated, and brain and spleen showed the highest ArgMet levels. In line with the specific pattern of ADMA, SDMA, and MMA observed in cells, ADMA was the most abundant methylated species, followed by SDMA and MMA. During aging of mice, levels of protein ArgMet changed drastically in brain and spleen proteins, whereas other tissues, such as heart, liver, and kidney, were less affected. The spleen is among the most affected organs during aging, and a link to the accumulation of senescent cells has been hypothesized (Lim et al., 2008, 2010). Thus, it is conceivable that the loss of protein ArgMet is associated with loss of PRMT1 expression/activity under physiological conditions, as demonstrated by a recent study linking PRMT1 downregulation with senescence of neuroblastoma cells (Lee et al., 2019). High levels of protein ArgMet in spleen and a strong reduction during aging raises the question as to whether accelerated aging of the spleen could be an inevitable side effect of the aforementioned protein arginine methyltransferase inhibitors. First links between ArgMet and neurodegenerative diseases have been suggested, as hypomethylated RNA-binding proteins FUS and poly-GR dipeptide repeats were found to be enriched in patients with frontotemporal dementia and amyotrophic lateral sclerosis, respectively (Dormann et al., 2012; Gittings et al., 2020; Suarez-Calvet et al., 2016). Given the globally reduced levels of SDMA/MMA in the brain of old mice, it will be interesting to investigate whether ArgMet is associated with the risk of neurodegenerative diseases, for example through modulation of LLPS of RNA-binding proteins.

Taken together, our findings support the idea that (1) protein ArgMet is a highly abundant PTM in cells and tissues, (2) ArgMet and specific aspects of metabolism are tightly coupled, (3) "demethylation" is a slow process, and (4) cancer and aging lead to substantial changes in global ArgMet levels. Given its relatively high proportion, we hypothesize that ArgMet plays a key role in maintaining cellular homeostasis, for example by regulating LLPS and formation of membrane-less organelles on a global scale. Concentrations of ADMA, SDMA, and MMA in proteins might be used as biomarkers for drug discovery, treatment response, and (potentially) diagnosis of tumor susceptibility for arginine methyltransferase inhibitors. These findings could lead to the development of improved methods for basic research on ArgMet and implementation of routine ArgMet-based screening in the clinic.

Limitations of the study

Despite the qualitative and quantitative information gathered by using our ArgMet-NMR protocol, some limitations to this study exist. With this assay, the detection limit for ADMA was approximately 100 nM (Figure S1H), and a saturation level of the SPE column of 3 mM arginine has been observed (Figure S1I). Thus, more material might be necessary for samples with low ArgMet content. Our method relies on sample clean-up by SPE using cation-exchange columns, which leads to a loss of some acidic and neutral amino acids and derivatives thereof. Distinguishing MMA from SDMA requires an additional analysis step using DMSO as solvent. In contrast to proteomics-based methods, our protocol does not provide site-specific ArgMet information. Nevertheless, our method could be combined with peptide-based libraries to evaluate sequence specific ArgMet mediated by PRMTs *in vitro*. Our protocol provides insight into changes in ArgMet levels related to cancers, cell differentiation, and aging. However, whether ArgMet is a cause or consequence in these contexts requires further studies in the future.

STAR★METHODS

Detailed methods are provided in the online version of this paper and include the following:

- KEY RESOURCES TABLE
- RESOURCE AVAILABILITY
  - Lead contact
  - Materials availability
  - Data and code availability
- EXPERIMENTAL MODEL AND SUBJECT DETAILS
  - Cell lines and culture conditions
  - Yeast strain and culture conditions
  - Organoid culture
  - Animals and diets
- METHOD DETAILS
  - *In vitro* methylation assay
  - Sample preparation
  - NMR measurements and spectral processing

- ○ High performance liquid chromatography (HPLC) assay
- ○ RNA isolation, reverse transcription and real-time PCR
- ● QUANTIFICATION AND STATISTICAL ANALYSIS

SUPPLEMENTAL INFORMATION

Supplemental information can be found online at https://doi.org/10.1016/j.crmeth.2021.100016.

ACKNOWLEDGMENTS

The work was supported by Austrian Science Fund (FWF) grants P28854, I3792, doc.funds BioMolStruct DOC 130 and DK-MCD W1226 to T.M., FWF grant P30162 to U.S., and FWF grant P33957 to T.E. B.P., T.M., and U.S. were supported by BioTechMed-Graz (Flagship project DYNIMO). T.M. was supported by the Austrian Research Promotion Agency (FFG) grants 864690 and 870454, the Integrative Metabolism Research Center Graz; Austrian Infrastructure Program 2016/2017, and the Styrian Government (Zukunftsfonds). D.K. was supported by the FWF (SFB F73, W1226, P32400, P30882, DP-iDP DOC 31), the BioTechMed-Graz flagship project "Lipases and Lipid Signaling," the County of Styria, and the City of Graz. We thank the Center of Medical Research for access to cell culture facilities, and Arno Absenger and Isabella Hindler for mouse care. F.Z. was trained within the frame of the PhD program Molecular Medicine, Medical University of Graz.

AUTHOR CONTRIBUTIONS

Conceptualization, T.M.; methodology, F.Z., J.K.-K., A.A., M.K., A.M., A.P., K.B.K., N.V., S.F., E.J.-L., G.H., M.P., M.J.R.C., M.P., M.M., T.E., B.P., and T.M.; Software, F.Z. and T.M.; validation, F.Z., T.M., A.M., and A.P.; formal analysis, F.Z. and T.M.; investigation, F.Z. and T.M.; resources, T.M., G.H., E.S., D.K., and U.S.; data curation, F.Z. and T.M.; writing – original draft, F.Z. and T.M.; writing – review and editing, F.Z., J.K.-K., A.A., M.K., K.B.K., N.V., T.M., E.S., G.H., D.K., U.S., M.J.R.C., B.B., T.E., B.R., B.P., M.P., M.M., A.M., and A.P.; visualization, T.M. and F.Z.; supervision, T.M.; project administration, T.M.; funding acquisition, T.M., D.K., U.S., B.P., and T.E. All authors have read and agreed to the published version of the manuscript.

DECLARATION OF INTERESTS

The authors declare no competing interests.

Received: January 7, 2021
Revised: April 2, 2021
Accepted: May 12, 2021
Published: June 21, 2021

REFERENCES

Afman, L.A., Blom, H.J., Drittij, M.-J., Brouns, M.R., and van Straaten, H.W. (2005). Inhibition of transmethylation disturbs neurulation in chick embryos. Dev. Brain Res. *158*, 59–65.

Bachand, F. (2007). Protein arginine methyltransferases: from unicellular eukaryotes to humans. Eukaryot. Cell *6*, 889–898.

Baker Brachmann, C., Davies, A., Cost, G.J., Caputo, E., Li, J., Hieter, P., and Boeke, J.D. (1998). Designer deletion strains derived from Saccharomyces cerevisiae S288C: a useful set of strains and plasmids for PCR-mediated gene disruption and other applications. Yeast *14*, 115–132.

Balint, B.L., Szanto, A., Madi, A., Bauer, U.-M., Gabor, P., Benko, S., Puskás, L.G., Davies, P.J., and Nagy, L. (2005). Arginine methylation provides epigenetic transcription memory for retinoid-induced differentiation in myeloid cells. Mol. Cell. Biol. *25*, 5648–5663.

Bárcena, C., Quirós, P.M., Durand, S., Mayoral, P., Rodríguez, F., Caravia, X.M., Mariño, G., Garabaya, C., Fernández-García, M.T., and Kroemer, G. (2018). Methionine restriction extends lifespan in progeroid mice and alters lipid and bile acid metabolism. Cell Rep. *24*, 2392–2403.

Bedford, M.T. (2007). Arginine methylation at a glance. J. Cell Sci. *120*, 4243–4246.

Bedford, M.T., and Clarke, S.G. (2009). Protein arginine methylation in mammals: who, what, and why. Mol. Cell *33*, 1–13.

Bedford, M.T., and Richard, S. (2005). Arginine methylation: an emerging regulator of protein function. Mol. Cell *18*, 263–272.

Bhatter, N., Roy, R., Shah, S., Sastry, S.P., Parbin, S., Iyappan, R., Kankaria, S., and Rajyaguru, P.I. (2019). Arginine methylation augments Sbp1 function in translation repression and decapping. FEBS J. *286*, 4693–4708.

Blanc, R.S., and Richard, S. (2017). Arginine methylation: the coming of age. Mol. Cell *65*, 8–24.

Blanc, R.S., Vogel, G., Chen, T., Crist, C., and Richard, S. (2016). PRMT7 preserves satellite cell regenerative capacity. Cell Rep. *14*, 1528–1539.

Boffa, L.C., Karn, J., Vidali, G., and Allfrey, V.G. (1977). Distribution of NG, NG,-dimethylarginine in nuclear protein fractions. Biochem. Biophys. Res. Commun. *74*, 969–976.

Bourgeois, B., Hutten, S., Gottschalk, B., Hofweber, M., Richter, G., Sternat, J., Abou-Ajram, C., Göbl, C., Leitinger, G., and Graier, W.F. (2020). Nonclassical nuclear localization signals mediate nuclear import of CIRBP. Proc. Natl. Acad. Sci. U S A *117*, 8503–8514.

Bremang, M., Cuomo, A., Agresta, A.M., Stugiewicz, M., Spadotto, V., and Bonaldi, T. (2013). Mass spectrometry-based identification and characterisation of lysine and arginine methylation in the human proteome. Mol. Biosyst. *9*, 2231–2247.

Bulau, P., Zakrzewicz, D., Kitowska, K., Wardega, B., Kreuder, J., and Eickelberg, O. (2006). Quantitative assessment of arginine methylation in free versus protein-incorporated amino acids in vitro and in vivo using protein hydrolysis and high-performance liquid chromatography. Biotechniques *40*, 305–310.

Chan-Penebre, E., Kuplast, K.G., Majer, C.R., Boriack-Sjodin, P.A., Wigle, T.J., Johnston, L.D., Rioux, N., Munchhof, M.J., Jin, L., and Jacques, S.L. (2015). A selective inhibitor of PRMT5 with in vivo and in vitro potency in MCL models. Nat. Chem. Biol. *11*, 432.

Chang, B., Chen, Y., Zhao, Y., and Bruick, R.K. (2007). JMJD6 is a histone arginine demethylase. Science *318*, 444–447.

Cheng, D., Cote, J., Shaaban, S., and Bedford, M.T. (2007). The arginine methyltransferase CARM1 regulates the coupling of transcription and mRNA processing. Mol. Cell *25*, 71–83.

Chern, M.-K., Chang, K.-N., Liu, L.-F., Tam, T.-C.S., Liu, Y.-C., Liang, Y.-L., and Tam, M.F. (2002). Yeast ribosomal protein L12 is a substrate of protein-arginine methyltransferase 2. J. Biol. Chem. *277*, 15345–15353.

Chia, S.Z., Lai, Y.W., Yagoub, D., Lev, S., Hamey, J.J., Pang, C.N.I., Desmarini, D., Chen, Z., Djordjevic, J.T., Erce, M.A., et al. (2018). Knockout of the Hmt1p arginine methyltransferase in *Saccharomyces cerevisiae* leads to the dysregulation of phosphate-associated genes and processes. Mol. Cell. Proteomics *17*, 2462–2479.

Chong, P.A., Vernon, R.M., and Forman-Kay, J.D. (2018). RGG/RG motif regions in RNA binding and phase separation. J. Mol. Biol. *430*, 4650–4665.

Counihan, J.L., Grossman, E.A., and Nomura, D.K. (2018). Cancer metabolism: current understanding and therapies. Chem. Rev. *118*, 6893–6923.

Curi, R., Lagranha, C.J., Doi, S.Q., Sellitti, D., Procópio, J., Pithon-Curi, T.C., Corless, M., and Newsholme, P. (2005). Molecular mechanisms of glutamine action. J. Cell. Physiol. *204*, 392–401.

Dhar, S., Vemulapalli, V., Patananan, A.N., Huang, G.L., Di Lorenzo, A., Richard, S., Comb, M.J., Guo, A., Clarke, S.G., and Bedford, M.T. (2013). Loss of the major Type I arginine methyltransferase PRMT1 causes substrate scavenging by other PRMTs. Sci. Rep. *3*, 1311.

Dormann, D., Madl, T., Valori, C.F., Bentmann, E., Tahirovic, S., Abou-Ajram, C., Kremmer, E., Ansorge, O., Mackenzie, I.R., Neumann, M., et al. (2012). Arginine methylation next to the PY-NLS modulates Transportin binding and nuclear import of FUS. EMBO J. *31*, 4258–4275.

Ducker, G.S., and Rabinowitz, J.D. (2017). One-carbon metabolism in health and disease. Cell Metab. *25*, 27–42.

Eram, M.S., Shen, Y., Szewczyk, M.M., Wu, H., Senisterra, G., Li, F., Butler, K.V., Kaniskan, H.U.m., Speed, B.A., and dela Sena, C. (2016). A potent, selective, and cell-active inhibitor of human type I protein arginine methyltransferases. ACS Chem. Biol. *11*, 772–781.

Erce, M.A., Abeygunawardena, D., Low, J.K., Hart-Smith, G., and Wilkins, M.R. (2013). Interactions affected by arginine methylation in the yeast protein-protein interaction network. Mol. Cell. Proteomics *12*, 3184–3198.

Esse, R., Imbard, A., Florindo, C., Gupta, S., Quinlivan, E.P., Davids, M., Teerlink, T., Tavares de Almeida, I., Kruger, W.D., Blom, H.J., et al. (2014). Protein arginine hypomethylation in a mouse model of cystathionine beta-synthase deficiency. FASEB J. *28*, 2686–2695.

Fedoriw, A., Rajapurkar, S.R., O'Brien, S., Gerhart, S.V., Mitchell, L.H., Adams, N.D., Rioux, N., Lingaraj, T., Ribich, S.A., and Pappalardi, M.B. (2019). Anti-tumor activity of the type I PRMT inhibitor, GSK3368715, synergizes with PRMT5 inhibition through MTAP loss. Cancer Cell *36*, 100–114.e25.

Feng, Y., Maity, R., Whitelegge, J.P., Hadjikyriacou, A., Li, Z., Zurita-Lopez, C., Al-Hadid, Q., Clark, A.T., Bedford, M.T., and Masson, J.-Y. (2013). Mammalian protein arginine methyltransferase 7 (PRMT7) specifically targets RXR sites in lysine-and arginine-rich regions. J. Biol. Chem. *288*, 37010–37025.

Fulton, M.D., Brown, T., and Zheng, Y.G. (2019). The biological axis of protein arginine methylation and asymmetric dimethylarginine. Int. J. Mol. Sci. *20*, 3322.

Gary, J.D., and Clarke, S. (1998). RNA and protein interactions modulated by protein arginine methylation. Prog. Nucleic Acid Res. Mol. Biol. *61*, 65–131.

Gary, J.D., Lin, W.J., Yang, M.C., Herschman, H.R., and Clarke, S. (1996). The predominant protein-arginine methyltransferase from *Saccharomyces cerevisiae*. J. Biol. Chem. *271*, 12585–12594.

Gelens, L., and Saurin, A.T. (2018). Exploring the function of dynamic phosphorylation-dephosphorylation cycles. Dev. Cell *44*, 659–663.

Gerhart, S.V., Kellner, W.A., Thompson, C., Pappalardi, M.B., Zhang, X.-P., de Oca, R.M., Penebre, E., Duncan, K., Boriack-Sjodin, A., and Le, B. (2018). Activation of the p53-MDM4 regulatory axis defines the anti-tumour response to PRMT5 inhibition through its role in regulating cellular splicing. Sci. Rep. *8*, 9711.

Gittings, L.M., Boeynaems, S., Lightwood, D., Clargo, A., Topia, S., Nakayama, L., Troakes, C., Mann, D.M.A., Gitler, A.D., Lashley, T., et al. (2020). Symmetric dimethylation of poly-GR correlates with disease duration in C9orf72 FTLD and ALS and reduces poly-GR phase separation and toxicity. Acta Neuropathol. *139*, 407–410.

Goulet, I., Gauvin, G., Boisvenue, S., and Côté, J. (2007). Alternative splicing yields protein arginine methyltransferase 1 isoforms with distinct activity, substrate specificity, and subcellular localization. J. Biol. Chem. *282*, 33009–33021.

Grandison, R.C., Piper, M.D., and Partridge, L. (2009). Amino-acid imbalance explains extension of lifespan by dietary restriction in *Drosophila*. Nature *462*, 1061–1064.

Guccione, E., and Richard, S. (2019). The regulation, functions and clinical relevance of arginine methylation. Nat. Rev. Mol. Cell Biol. *20*, 642–657.

Guo, A., Gu, H., Zhou, J., Mulhern, D., Wang, Y., Lee, K.A., Yang, V., Aguiar, M., Kornhauser, J., and Jia, X. (2014). Immunoaffinity enrichment and mass spectrometry analysis of protein methylation. Mol. Cell. Proteomics *13*, 372–387.

Haber, A.L., Biton, M., Rogel, N., Herbst, R.H., Shekhar, K., Smillie, C., Burgin, G., Delorey, T.M., Howitt, M.R., Katz, Y., et al. (2017). A single-cell survey of the small intestinal epithelium. Nature *551*, 333–339.

Hamamoto, R., and Nakamura, Y. (2016). Dysregulation of protein methyltransferases in human cancer: an emerging target class for anticancer therapy. Cancer Sci. *107*, 377–384.

Hamey, J.J., Rakow, S., Bouchard, C., Senst, J.M., Kolb, P., Bauer, U.-M., Wilkins, M.R., and Hart-Smith, G. (2021). Systematic investigation of PRMT6 substrate recognition reveals broad specificity with a preference for an RG motif or basic and bulky residues. FEBS J. https://doi.org/10.1111/febs.15837.

Hartel, N.G., Chew, B., Qin, J., Xu, J., and Graham, N.A. (2019). Deep protein methylation profiling by combined chemical and immunoaffinity approaches reveals novel PRMT1 targets. Mol. Cell. Proteomics *18*, 2149–2164.

Henry, M.F., and Silver, P.A. (1996). A novel methyltransferase (Hmt1p) modifies poly(A)$^+$-RNA-binding proteins. Mol. Cell Biol. *16*, 3668–3678.

Herrmann, F., Pably, P., Eckerich, C., Bedford, M.T., and Fackelmayer, F.O. (2009). Human protein arginine methyltransferases in vivo—distinct properties of eight canonical members of the PRMT family. J. Cell Sci. *122*, 667–677.

Hofweber, M., Hutten, S., Bourgeois, B., Spreitzer, E., Niedner-Boblenz, A., Schifferer, M., Ruepp, M.-D., Simons, M., Niessing, D., and Madl, T. (2018). Phase separation of FUS is suppressed by its nuclear import receptor and arginine methylation. Cell *173*, 706–719.e13.

Hong, E., Lim, Y., Lee, E., Oh, M., and Kwon, D. (2012). Tissue-specific and age-dependent expression of protein arginine methyltransferases (PRMTs) in male rat tissues. Biogerontology *13*, 329–336.

Hornbeck, P.V., Zhang, B., Murray, B., Kornhauser, J.M., Latham, V., and Skrzypek, E. (2015). PhosphoSitePlus, 2014: mutations, PTMs and recalibrations. Nucleic Acids Res. *43*, D512–D520.

Hsieh, C.H., Huang, S.Y., Wu, Y.C., Liu, L.F., Han, C.C., Liu, Y.C., and Tam, M.F. (2007). Expression of proteins with dimethylarginines in *Escherichia coli* for protein-protein interaction studies. Protein Sci. *16*, 919–928.

Jarrold, J., and Davies, C.C. (2019). PRMTs and arginine methylation: cancer's best-kept secret? Trends Mol. Med. *25*, 993–1009.

Kuilman, T., Michaloglou, C., Vredeveld, L.C., Douma, S., van Doorn, R., Desmet, C.J., Aarden, L.A., Mooi, W.J., and Peeper, D.S. (2008). Oncogene-induced senescence relayed by an interleukin-dependent inflammatory network. Cell *133*, 1019–1031.

Kusevic, D., Kudithipudi, S., and Jeltsch, A. (2016). Substrate specificity of the HEMK2 protein glutamine methyltransferase and identification of novel substrates. J. Biol. Chem. *291*, 6124–6133.

Larsen, S.C., Sylvestersen, K.B., Mund, A., Lyon, D., Mullari, M., Madsen, M.V., Daniel, J.A., Jensen, L.J., and Nielsen, M.L. (2016). Proteome-wide analysis of arginine monomethylation reveals widespread occurrence in human cells. Sci. Signal. *9*, rs9.

Lee, B.C., Kaya, A., and Gladyshev, V.N. (2016). Methionine restriction and lifespan control. Ann. N Y Acad. Sci. *1363*, 116.

Lee, J., and Bedford, M.T. (2002). PABP1 identified as an arginine methyltransferase substrate using high-density protein arrays. EMBO Rep. *3*, 268–273.

Lee, J., Sayegh, J., Daniel, J., Clarke, S., and Bedford, M.T. (2005). PRMT8, a new membrane-bound tissue-specific member of the protein arginine methyltransferase family. J. Biol. Chem. *280*, 32890–32896.

Lee, Y.-J., Chang, W.-W., Chang, C.-P., Liu, T.-Y., Chuang, C.-Y., Qian, K., Zheng, Y.G., and Li, C. (2019). Downregulation of PRMT1 promotes the senescence and migration of a non-MYCN amplified neuroblastoma SK-N-SH cells. Sci. Rep. *9*, 1771.

Lee, Y.H., and Stallcup, M.R. (2009). Minireview: protein arginine methylation of nonhistone proteins in transcriptional regulation. Mol. Endocrinol. *23*, 425–433.

Li, L., Zhang, Z., Ma, T., and Huo, R. (2016). PRMT1 regulates tumor growth and metastasis of human melanoma via targeting ALCAM. Mol. Med. Rep. *14*, 521–528.

Lim, Y., Hong, E., Kwon, D., and Lee, E. (2010). Proteomic identification and comparative analysis of asymmetrically arginine-methylated proteins in immortalized, young and senescent cells. Electrophoresis *31*, 3823–3833.

Lim, Y., Lee, E., Lee, J., Oh, S., and Kim, S. (2008). Down-regulation of asymmetric arginine methylation during replicative and H_2O_2-induced premature senescence in WI-38 human diploid fibroblasts. J. Biochem. *144*, 523–529.

Lindeboom, R.G., van Voorthuijsen, L., Oost, K.C., Rodríguez-Colman, M.J., Luna-Velez, M.V., Furlan, C., Baraille, F., Jansen, P.W., Ribeiro, A., and

Burgering, B.M. (2018). Integrative multi-omics analysis of intestinal organoid differentiation. Mol. Syst. Biol. *14*, e8227.

Litt, M., Qiu, Y., and Huang, S. (2009). Histone arginine methylations: their roles in chromatin dynamics and transcriptional regulation. Biosci. Rep. *29*, 131–141.

Liu, F., Cheng, G., Hamard, P.-J., Greenblatt, S., Wang, L., Man, N., Perna, F., Xu, H., Tadi, M., and Luciani, L. (2015). Arginine methyltransferase PRMT5 is essential for sustaining normal adult hematopoiesis. J. Clin. Invest. *125*, 3532–3544.

Liu, Y.J., Janssens, G.E., McIntyre, R.L., Molenaars, M., Kamble, R., Gao, A.W., Jongejan, A., Weeghel, M.v., MacInnes, A.W., and Houtkooper, R.H. (2019). Glycine promotes longevity in *Caenorhabditis elegans* in a methionine cycle-dependent fashion. PLoS Genet. *15*, e1007633.

Locasale, J.W. (2013). Serine, glycine and one-carbon units: cancer metabolism in full circle. Nat. Rev. Cancer *13*, 572–583.

Lorton, B.M., and Shechter, D. (2019). Cellular consequences of arginine methylation. Cell Mol. Life Sci. *76*, 2933–2956.

Low, J.K., Im, H., Erce, M.A., Hart-Smith, G., Snyder, M.P., and Wilkins, M.R. (2016). Protein substrates of the arginine methyltransferase Hmt1 identified by proteome arrays. Proteomics *16*, 465–476.

Low, J.K., and Wilkins, M.R. (2012). Protein arginine methylation in *Saccharomyces cerevisiae*. FEBS J. *279*, 4423–4443.

Ludikhuize, M.C., Meerlo, M., Gallego, M.P., Xanthakis, D., Burgaya Julia, M., Nguyen, N.T.B., Brombacher, E.C., Liv, N., Maurice, M.M., Paik, J.H., et al. (2020). Mitochondria define intestinal stem cell differentiation downstream of a FOXO/Notch axis. Cell Metab. *32*, 889–900.e7.

Luka, Z., Mudd, S.H., and Wagner, C. (2009). Glycine N-methyltransferase and regulation of S-adenosylmethionine levels. J. Biol. Chem. *284*, 22507–22511.

Martinez, A., Bourdreux, F., Riguet, E., and Nuzillard, J.M. (2012). High-resolution and high-sensitivity 2D homonuclear J-resolved NMR spectroscopy. Magn. Reson. Chem. *50*, 28–32.

Matsuoka, M. (1972). [Epsilon-N-methylated lysine and guanidine-N-methylated arginine of proteins. 3. Presence and distribution in nature and mammals]. Seikagaku *44*, 364–370.

Meinitzer, A., Puchinger, M., Winklhofer-Roob, B.M., Rock, E., Ribalta, J., Roob, J.M., Sundl, I., Halwachs-Baumann, G., and März, W. (2007). Reference values for plasma concentrations of asymmetrical dimethylarginine (ADMA) and other arginine metabolites in men after validation of a chromatographic method. Clinica Chim. Acta *384*, 141–148.

Mentch, S.J., and Locasale, J.W. (2016). One carbon metabolism and epigenetics: understanding the specificity. Ann. N Y Acad. Sci. *1363*, 91.

Miranda, T.B., Sayegh, J., Frankel, A., Katz, J.E., Miranda, M., and Clarke, S. (2006). Yeast Hsl7 (histone synthetic lethal 7) catalyses the in vitro formation of omega-N(G)-monomethylarginine in calf thymus histone H2A. Biochem. J. *395*, 563–570.

Musiani, D., Bok, J., Massignani, E., Wu, L., Tabaglio, T., Ippolito, M.R., Cuomo, A., Ozbek, U., Zorgati, H., and Ghoshdastider, U. (2019). Proteomics profiling of arginine methylation defines PRMT5 substrate specificity. Sci. Signal. *12*, eaat8388.

Musiani, D., Giambruno, R., Massignani, E., Ippolito, M.R., Maniaci, M., Jammula, S., Manganaro, D., Cuomo, A., Nicosia, L., and Pasini, D. (2020). PRMT1 is recruited via DNA-PK to chromatin where it sustains the senescence-associated secretory phenotype in response to Cisplatin. Cell Rep. *30*, 1208–1222.e9.

Nagayama, K., Wüthrich, K., Bachmann, P., and Ernst, R.R. (1977). Two-dimensional J-resolved ^{1}H n.m.r. spectroscopy for studies of biological macromolecules. Biochem. Biophys. Res. Commun. *78*, 99–105.

Niewmierzycka, A., and Clarke, S. (1999). S-Adenosylmethionine-dependent methylation in *Saccharomyces cerevisiae*. Identification of a novel protein arginine methyltransferase. J. Biol. Chem. *274*, 814–824.

Nuzillard, J.-M. (1996). Time-reversal of NMR signals by linear prediction. application to phase-sensitive homonuclear J-resolved spectroscopy. J. Magn. Reson. *118*, 132–135.

Pahlich, S., Zakaryan, R.P., and Gehring, H. (2006). Protein arginine methylation: cellular functions and methods of analysis. Biochim. Biophys. Acta *1764*, 1890–1903.

Paik, W.K., and Kim, S. (1967). Enzymatic methylation of protein fractions from calf thymus nuclei. Biochem. Biophys. Res. Commun. *29*, 14–20.

Paik, W.K., Paik, D.C., and Kim, S. (2007). Historical review: the field of protein methylation. Trends Biochem. Sci. *32*, 146–152.

Partridge, L., Fuentealba, M., and Kennedy, B.K. (2020). The quest to slow ageing through drug discovery. Nat. Rev. Drug Discov. *19*, 513–532.

Pawlak, M.R., Scherer, C.A., Chen, J., Roshon, M.J., and Ruley, H.E. (2000). Arginine N-methyltransferase 1 is required for early postimplantation mouse development, but cells deficient in the enzyme are viable. Mol. Cell Biol. *20*, 4859–4869.

Plank, M., Fischer, R., Geoghegan, V., Charles, P.D., Konietzny, R., Acuto, O., Pears, C., Schofield, C.J., and Kessler, B.M. (2015). Expanding the yeast protein arginine methylome. Proteomics *15*, 3232–3243.

Sakhaii, P., and Bermel, W. (2014). Improving the sensitivity of conventional spin echo spectra by preservation of initial signal-to-noise ratio. J. Magn. Reson. *242*, 220–223.

Sayegh, J., and Clarke, S.G. (2008). Hsl7 is a substrate-specific type II protein arginine methyltransferase in yeast. Biochem. Biophys. Res. Commun. *372*, 811–815.

Scorilas, A., Black, M.H., Talieri, M., and Diamandis, E.P. (2000). Genomic organization, physical mapping, and expression analysis of the human protein arginine methyltransferase 1 gene. Biochem. Biophys. Res. Commun. *278*, 349–359.

Shishkova, E., Zeng, H., Liu, F., Kwiecien, N.W., Hebert, A.S., Coon, J.J., and Xu, W. (2017). Global mapping of CARM1 substrates defines enzyme specificity and substrate recognition. Nat. Commun. *8*, 15571.

Stopa, N., Krebs, J.E., and Shechter, D. (2015). The PRMT5 arginine methyltransferase: many roles in development, cancer and beyond. Cell Mol. Life Sci. *72*, 2041–2059.

Stryeck, S., Horvath, A., Leber, B., Stadlbauer, V., and Madl, T. (2018). NMR spectroscopy enables simultaneous quantification of carbohydrates for diagnosis of intestinal and gastric permeability. Sci. Rep. *8*, 14650.

Suarez-Calvet, M., Neumann, M., Arzberger, T., Abou-Ajram, C., Funk, E., Hartmann, H., Edbauer, D., Kremmer, E., Gobl, C., Resch, M., et al. (2016). Monomethylated and unmethylated FUS exhibit increased binding to Transportin and distinguish FTLD-FUS from ALS-FUS. Acta Neuropathol. *131*, 587–604.

Tang, J., Frankel, A., Cook, R.J., Kim, S., Paik, W.K., Williams, K.R., Clarke, S., and Herschman, H.R. (2000). PRMT1 is the predominant type I protein arginine methyltransferase in mammalian cells. J. Biol. Chem. *275*, 7723–7730.

Tee, W.W., Pardo, M., Theunissen, T.W., Yu, L., Choudhary, J.S., Hajkova, P., and Surani, M.A. (2010). Prmt5 is essential for early mouse development and acts in the cytoplasm to maintain ES cell pluripotency. Genes Dev. *24*, 2772–2777.

Thandapani, P., O'Connor, T.R., Bailey, T.L., and Richard, S. (2013). Defining the RGG/RG motif. Mol. Cell *50*, 613–623.

Uhlen, M., Fagerberg, L., Hallstrom, B.M., Lindskog, C., Oksvold, P., Mardinoglu, A., Sivertsson, A., Kampf, C., Sjostedt, E., Asplund, A., et al. (2015). Proteomics. Tissue-based map of the human proteome. Science *347*, 1260419.

Uhlmann, T., Geoghegan, V.L., Thomas, B., Ridlova, G., Trudgian, D.C., and Acuto, O. (2012). A method for large-scale identification of protein arginine methylation. Mol. Cell. Proteomics *11*, 1489–1499.

Vander Heiden, M.G., and DeBerardinis, R.J. (2017). Understanding the intersections between metabolism and cancer biology. Cell *168*, 657–669.

Viant, M.R., Rosenblum, E.S., and Tieerdema, R.S. (2003). NMR-based metabolomics: a powerful approach for characterizing the effects of environmental stressors on organism health. Environ. Sci. Technol. *37*, 4982–4989.

Wang, Y., Bollard, M.E., Keun, H., Antti, H., Beckonert, O., Ebbels, T.M., Lindon, J.C., Holmes, E., Tang, H., and Nicholson, J.K. (2003). Spectral editing

CellPress
OPEN ACCESS

and pattern recognition methods applied to high-resolution magic-angle spinning ^{1}H nuclear magnetic resonance spectroscopy of liver tissues. Anal Biochem. *323*, 26–32.

Wang, Y., Wysocka, J., Sayegh, J., Lee, Y.H., Perlin, J.R., Leonelli, L., Sonbuchner, L.S., McDonald, C.H., Cook, R.G., Dou, Y., et al. (2004). Human PAD4 regulates histone arginine methylation levels via demethylimination. Science *306*, 279–283.

Wang, Y.-C., Wang, C.-W., Lin, W.-C., Tsai, Y.-J., Chang, C.-P., Lee, Y.-J., Lin, M.-J., and Li, C. (2017). Identification, chromosomal arrangements and expression analyses of the evolutionarily conserved prmt1 gene in chicken in comparison with its vertebrate paralogue prmt8. PLoS One *12*, e0185042.

Wooderchak, W.L., Zang, T., Zhou, Z.S., Acuna, M., Tahara, S.M., and Hevel, J.M. (2008). Substrate profiling of PRMT1 reveals amino acid sequences that extend beyond the "RGG" paradigm. Biochemistry *47*, 9456–9466.

Woodsmith, J., Casado-Medrano, V., Benlasfer, N., Eccles, R.L., Hutten, S., Heine, C.L., Thormann, V., Abou-Ajram, C., Rocks, O., and Dormann, D. (2018). Interaction modulation through arrays of clustered methyl-arginine protein modifications. Life Sci. Alliance *1*, e201800178.

Yang, M., and Vousden, K.H. (2016). Serine and one-carbon metabolism in cancer. Nat. Rev. Cancer *16*, 650–662.

Yang, Y., and Bedford, M.T. (2013). Protein arginine methyltransferases and cancer. Nat. Rev. Cancer *13*, 37–50.

Young, B.D., Weiss, D.I., Zurita-Lopez, C.I., Webb, K.J., Clarke, S.G., and McBride, A.E. (2012). Identification of methylated proteins in the yeast small ribosomal subunit: a role for SPOUT methyltransferases in protein arginine methylation. Biochemistry *51*, 5091–5104.

STAR★METHODS

KEY RESOURCES TABLE

REAGENT or RESOURCE	SOURCE	IDENTIFIER
Bacterial and virus strains		
E. coli BL21-DE3 Star strain	Agilent Technologies	CAT#: 200131; Lot#: 0006276950
Chemicals, peptides, and recombinant proteins		
L-arginine	AppliChem	Cat # A3675
ω-NG, NG-asymmetric dimethylarginine (ADMA)	Santa Cruz Biotechnology	Cat # sc-208093
ω-NG-N'G-symmetric dimethylarginine (SDMA)	Santa Cruz Biotechnology	Cat # sc-202235A
ω-NG-monomethylarginine (MMA)	Santa Cruz Biotechnology	Cat # sc-200739A
Sodium phosphate, dibasic (Na_2HPO_4)	VWR	Cat # 80731-078
3-(trimethylsilyl) propionic acid-2,2,3,3-d4 sodium salt (TSP)	Alfa Aesar	Cat # A1448
sodium hydroxide	VWR	Cat # BDH7363-4
Chloroform	VWR	Cat # MK444410
hydrochloric acid (32% m/v)	VWR	Cat # EM1.00313.2500
deuterium oxide (^2H_2O)	Cambridge Isotope laboratories	Cat # DLM-6-1000
DIMETHYL SULFOXIDE-D6 (d6-DMSO)	Cambridge Isotope laboratories	Cat # DLM-10-PK
Adenosine, periodate oxidized (AdOx)	Sigma Aldrich	Cat # A7154
MS023 hydrochloride	Sigma Aldrich	Cat # SML1555
GSK3203591	MedChemExpress Austria	Cat # HY-100235
GSK3368715 dihydrochloride	MedChemExpress Austria	Cat # HY-128717A
Methanol	Roth	Cat # 8388.4
Ammonia solution	Roth	Cat # A990.1
S-Adenosyl methionine	Biolabs	Cat # 10079762
L-Methionine-(methyl-^{13}C)	MERCK	Cat # 299146
BD Difco^{TM} Yeast Nitrogen Base without Amino Acids and Ammonium Sulfate	BD Biosciences	Cat # 233520
Ammonium sulphate	Roth	Cat # 3746.1
Uracil	Sigma Aldrich	Cat # U0750
Adenine	Serva	Cat # 10739
Amino Acids, Analytical grade	Serva	Cat # 11482, 13940, 14180, 14110, 17880, 22942, 23000, 23390, 24842, 26540, 27690, 28220, 28821, 32191, 33582, 34962, 36382, 37422, 37540, 38064
Glucose	PanReac, AppliChem	Cat # 143140.0914
Critical commercial assays		
Chromatin Extraction Kit	Abcam	Cat # ab117152
High Capacity cDNA Reverse Transcription Kit	Applied Biosystems	Cat # 4368814
Deposited data		
Mouse small intestine single cell RNAseq data	NCBI GEO	GEO: GSE92332
Experimental models: cell lines		
MDA-MB-231	Sigma Aldrich	Cat # 92020423
HaCat	ATCC	Cat # CRL-4048

(*Continued on next page*)

CellPress
OPEN ACCESS

Continued

REAGENT or RESOURCE	SOURCE	IDENTIFIER
MCF10A	LGC Promochem	Cat # ATCC-CRL-10317
A375	ATCC	Cat # CRL-1619
SW-872	ATCC	Cat # HTB-92
93T449	ATCC	Cat # CRL-3043
SW1353	CLS	Cat # 300440
juvenile fibroblasts	Division of Biomedical Research(BMF),Medical University of Graz	N/A
HeLa	ATCC	Cat # CCL-2
S. cerevisiae BY4741 (*MATa his3Δ-1 leu2Δ -0 met15Δ -0 ura3Δ -0*) wild type yeast	Euroscarf (http://www.euroscarf.de/)	Y00000
S. cerevisiae BY4742 (*MATα his3Δ-1 leu2Δ-0 lys2Δ-0 ura3Δ-0*) wild type yeast	Euroscarf (http://www.euroscarf.de/)	Y10000
S. cerevisiae BY4741 *Δhmt1* (BY4741 *ybr034c*::*kanMX4*)	Euroscarf (http://www.euroscarf.de/)	Y03171
S. cerevisiae BY4742 *Δhmt1* (BY4742 *ybr034c*::*kanMX4*)	Euroscarf (http://www.euroscarf.de/)	Y13171
S. cerevisiae BY4741 *Δhsl7* (BY4741 *ybr133c*::*kanMX4*)	Euroscarf (http://www.euroscarf.de/)	Y07539
S. cerevisiae BY4742 *Δhsl7* (BY4742 *ybr133c*::*kanMX4*)	Euroscarf (http://www.euroscarf.de/)	Y17539
Experimental models: organisms/strains		
Mouse: Mixed genetic background of 129/J and C57BL/6J	Gift from Dr. Dennis E. Vance (University of Alberta, Canada)	N/A
Mouse small intestinal organoid	Gift from Dr. Burgering	N/A
Oligonucleotides		
Primers for real-time PCR, see Table S1	This manuscript	N/A
Recombinant DNA		
Plasmid: CIRBP-RGG	Genscript	N/A
Plasmid: FUS-RGG-PY	Genscript	N/A
Plasmid:PRMT1	Genscript	N/A
Software and algorithms		
TopSpin^{TM} 4.0.6	Bruker	https://www.bruker.com
Chenomx Profiler nmr suite 8.4	Chenomx Inc	https://www.chenomx.com/
MestReNova 12.0.4-22023	Mestrelab Research S.L.	http://www.mestrelab.com

RESOURCE AVAILABILITY

Lead contact

Further information and requests for resources and reagents should be directed to and will be fulfilled by the Lead Contact, Tobias Madl (tobias.madl@medunigraz.at).

Materials availability

This study did not generate new unique reagents.

Data and code availability

This study did not generate computer algorithm or code. The article includes all data generated or analyzed during this study. Original source data for figures in the paper are available upon request to the lead contact author.

EXPERIMENTAL MODEL AND SUBJECT DETAILS

Cell lines and culture conditions

MDA-MB-231 (Sigma Aldrich, Vienna, Austria), HaCat (ATCC, US), MCF10A (LGC Promochem, US), A375 (ATCC, US), SW-872 (ATCC, US), 93T449 (ATCC, US), SW1353 (CLS, Germany) and juvenile fibroblasts fresh established from foreskin samples were obtained from Division of Biomedical Research (BMF), Medical University of Graz, Austria. HeLa (ATCC®, Guernsey, UK), fibroblast, HaCat, SW1353 and A375 cells were cultured in DMEM supplemented with 2 mM glutamine, 1% PS (100 U/mL penicillin, 100 μg/mL streptomycin) and 10% fetal bovine serum (FBS). MCF10A were cultured in DMEM with single quot kit suppl. Gr, 5% Horse Serum, 20 ng/mL hEGF, 0.5 μg/ml hydrocortison, 100 ng/ml choleratoxin, 10 μg/ml insulin and 2 mM glutamine. MDA-MB-231 were maintained in DMEM Hams F12 with 10% FBS, 2 mM glutamine and 1% PS. SW872 were cultured in DMEM Hams F12 supplemented with 5% FBS, 2 mM glutamine and 1% PS. 93T449 were cultured with RPMI-1640 with 10% FBS, 2 mM glutamine, 10mM 4-(2-hydroxyethyl)-1-piperazineethanesulfonic acid (HEPES), 1mM sodium pyruvate and 1% PS.

Cells were maintained in a humidified incubator at 37°C with 5% CO_2. HeLa cells were treated for up to 3 days with AdOx (40 μM), MS023 (10 μM), GSK715 (2 μM), GSK591 (1 μM) and DMSO, before cell extracts were prepared.

Yeast strain and culture conditions

Yeast experiments were carried out in *S. cerevisiae* BY4741 (*MATa his3Δ-1 leu2Δ-0 met15Δ-0 ura3Δ-0*) and BY4742 (*MATa his3Δ-1 leu2Δ-0 lys2Δ-0 ura3Δ-0*) wild type yeast (Baker Brachmann et al., 1998) and the same strains carrying either an *HMT1*-knockout (*hmt1*:kanMX4), or *HSL7*-knockout (*hsl7*:kanMX4) all obtained from Euroscarf. Correct presence of respective gene knockouts was verified by PCR using forward primers 5'-TGAAGACATCCCATGTCCAG-3' (HMT1_up), 5'-TGAATGCTACTGATGTCTGC-3' (HSL7_up), and reverse primer 5'-CAAGACTGTCAAGGAGGG- 3' (KanR5b). Cells were grown to logarithmic phase in SC 2% glucose medium consisting of 0.14% yeast nitrogen base (BD Difco^{TM}, 233520), 5% $(NH_4)_2SO_4$ supplemented with 30 mg/L of all amino acids (except 80 mg/L histidine, 200 mg/L leucine, 120 mg/L lysine and 26 mg/L methionine), 30 mg/L adenine, and 320 mg/L uracil, allowing comparable growth of both BY4741 and BY4742 strains. Fresh overnight cultures were diluted to 0.1 OD600 (Genesys 10uv photometer, corresponds to $\sim 2\times10^6$ cells/mL), incubated for 6 h at 28 °C, 145 rpm, to reach logarithmic growth phase at a culture density of ~0.6 OD600. 15 OD600 equivalents were harvested by centrifugation (1,700 g, 3 min, 4 °C), washed once with 10 ml ice-cold water, and the cell pellet was immediately snap frozen in liquid nitrogen and stored at -80 °C until processing for NMR analysis.

Organoid culture

Mouse small intestinal organoids were cultured as described previously (Lindeboom et al., 2018). In short, the organoids were maintained using basic culture (ENR) medium, which contained advanced DMEM/F12 supplemented with penicillin/streptomycin (1%, 10 mM HEPES, 1× Glutamax, 1× B27 (all from Life Technologies) and 1 mM N-acetylcysteine (Sigma) supplemented with murine recombinant epidermal growth factor (Peprotech), R-spondin1-CM (5% v/v) and noggin-CM (10% v/v). A mycoplasma-free status was confirmed routinely. Organoids were split every 4–5 days by mechanical disruption and plated in Matrigel. Three days after splitting, stem cell-enriched organoid cultures (CV) were generated by supplementation of ENR with CHIR99021 (3 μM) and valproic acid (1 mM). Paneth cell-enriched organoids were generated by addition of Chir (3 μM) and DAPT (5 μM), stem cell-depleted organoid cultures (EN) were grown in ENR medium without R-Spondin-1. Organoids were harvested after 3 days by using mechanical dissociation of matrigel followed by 3 washing steps with ice-cold PBS. Organoid pellets were immediately frozen at -80°C for further analysis.

Animals and diets

For all experiments, young (9-11 weeks) and old (96-104 weeks) female wild type mice (mixed genetic background of 129/J and C57BL/6J) were used (n=5). Mice were maintained in a clean, temperature-controlled (22 ± 1°C) environment with a regular light–dark cycle (12 h/12 h) and unlimited access to chow diet (Altromin 1324, Altromin Spezialfutter GmbH, Lage, Germany) and water. All experiments were performed in accordance with the European Directive 2010/63/EU and approved by the Austrian Federal Ministry of Education, Science and Research (GZ 66.010/0051-WF/V/3b/2015).

METHOD DETAILS

In vitro methylation assay

The recombinant CIRBP-RGG and FUS-RGG-PY sequences were as follows: RSRGYRGGSAGGRGFFRGGRGRGRGFSRGGGDRGYGG and GPGGGPGGSHMGGNYGDDRRGGRGGYDRGGYRGRGGDRGGFRGGRGGGDRGGFGPGKMDSRGEHRQDRRERPY. Expression and purification of recombinant His_6-PRMT1, His_6-CIRBP-RGG and His_6-FUS-RGG-PY have been described in previous study (Bourgeois et al., 2020; Hofweber et al., 2018). Untagged CIRBP-RGG and FUS-RGG-PY recombinant proteins and His_6-PRMT1 were equilibrated in methylation buffer containing 50 mM Tris-HCl, 150 mM NaCl, and 2 mM Tris(2-carboxyethyl)phosphine, pH 7.5; 100 μM CIRBP-RGG or FUS-RGG-PY was incubated with 10 μM His_6-PRMT1 in the presence of 2 mM S-Adenosylmethionine (New England Biolabs) for 16 h at room temperature. Untagged methylated CIRBPRGG (meCIRBP) and FUS-RGG-PY (meFUS) were then isolated from PRMT1 performing a second affinity purification using Ni-NTA beads, and further analyzed using NMR.

Sample preparation

Cells (5 x 10^6) were plated onto 60 mm dishes and incubated under standard conditions as described above. To harvest the cells, medium was removed, cells were washed three times with 5 mL of cold phosphate-buffered saline (PBS, 137 mM NaCl, 2.7 mM KCl, 8 mM Na_2HPO_4, and 2 mM KH_2PO_4) solution and collected using a cell scraper. A solution of 5 × 10^6 cells was centrifuged at 1,000 rpm for 1 min, the supernatant was discarded and the cell pellet was flash frozen in liquid nitrogen and stored at −80°C for the extraction step. The organs were isolated from sacrificed mice, divided into 20–30 mg and snap-frozen in liquid nitrogen for storage at -80°C until extraction. Cell pellets, tissues and mouse small intestinal organoids were re-suspended in 400 μl ice-cold methanol (-20°C) and 200 μl MilliQ H_2O and transferred to a tube containing Precellys beads (1.4 mm zirconium oxide beads, Bertin Technologies, Villeurbanne, France) for homogenization on a Precellys 24 homogeniser for 2 cycles of 20 seconds with 5,000 rpm, 10-s breaks. Cell and tissues debris were pelleted by centrifugation at 13,000 rpm for 30 min (4°C) and the precipitate was used for hydrolysis. Supernatants were frozen at −80°C and be used for e.g. metabolite analysis.

The precipitates were hydrolysed with 500 μl 9 M HCl for 12 h at 110°C to obtain (modified) amino acids. The solution was lyophilised and resuspended in 900 μl of 0.1 M HCl and 100 μl chloroform to remove lipids, centrifuged (10 min, 13,000 rpm) and the supernatant subjected to i) solid-phase-extraction (SPE) using Waters™ cartridges (1 mL Oasis MCX 1 cc/30 mg, Waters™, Eschborn, Germany) containing a mixed-mode polymeric sorbent with both reverse phase and cation exchange functionalities. Each step was performed with 1 mL of solution and by centrifugation at room temperature (1,000 rpm for 1 min). ii) auto SPE using Gilson® GX-241 ASPEC system (Gilson Incorporated, Middleton, WI) and Waters™ cartridges. The flow rate for the injection of liquids was set to 2 mL/min for the sample, 7 mL/min for the replacement solution and the 0.1 M HCl, and to 10 mL/min for methanol, PBS and MilliQ-water. The cartridges were pre-conditioned with a detachment solution (2x 1 mL, 10% NH_3 saturated solution, 40 % MilliQ H_2O, 50 % methanol), methanol (1x 1 mL) and with PBS (2x 1 mL). After sample loading (1x 1 mL), cartridges were washed with MilliQ-water (3x 1 mL), 0.1 M HCl (5x 1 mL) and methanol (2x 1 mL,). The arginine and its derivatives were recovered with the replacement solution (2x 1 mL), lyophylized and dissolved in 500 μl NMR buffer [0.08 M Na_2HPO_4, 5 mM 3-(trimethylsilyl) propionic acid-2,2,3,3-d4 sodium salt (TSP), 0.04 (w/v) % NaN_3 in D_2O, pH adjusted to 7.4 with 8 M HCl and 5 M NaOH] for measuring. The Chromatin Extraction Kit (ab117152, Abcam) was used for HeLa/A375 chromatin and non-chromatin fractions extraction according to the manufacturer's instructions.

NMR measurements and spectral processing

All NMR experiments were acquired at 310 K using Bruker 600 MHz spectrometer equipped with a TXI probe head. The 1D CPMG (Carr–Purcell–Meiboom–Gill) pulse sequence (cpmgpr1d, 512 scans, size of fid 73728, 11904.76 Hz spectral width, recycle delay 4 s), with water signal suppression using presaturation, was recorded for ^1H 1D NMR experiments. ^1H-^{13}C HSQC (heteronuclear single quantum coherence spectroscopy) NMR spectra were recorded for 13C-methyl labelled methionine assays with a recycle delay of 1.0 s, spectral widths of 20.8228/83.8554 ppm, centered at 3.923/50 ppm in ^1H/^{13}C, with 2048 and 256 points, respectively, and 8 scans per increment. The 2D JRES (^1H homo-nuclear J-resolved spectroscopy) pulse sequence (jresgpprqf, 16 scans, size of fid 16384 (direct dimension F2)/256 (indirect dimension F1), 10000.00/78.042 Hz spectral width in F2 (chemical shift axis)/F1 (spin-spin coupling axis), recycle delay 2 s, Figure S1J) with presaturation during the relaxation delay was recorded to obtain virtually decoupled spectra (Nagayama et al., 1977; Stryeck et al., 2018; Viant et al., 2003; Wang et al., 2003). In brief, data were processed in Bruker Topspin version 4.0.6 using one-dimensional exponential window multiplication of the FID, Fourier transformation and phase correction. Processing of 2D JRES was done using the SINE and QSINE window functions (SSB = 0) in F2/F1. Fourier transform was performed with 16384/256 F2/F1 points of the fid. 2D J-resolved experiments were processed using back prediction implemented in the Bruker au program proc_jres.be (Martinez et al., 2012; Nuzillard, 1996; Sakhaii and Bermel, 2014; Stryeck et al., 2018). The JRES spectra were then projected along F2 and exported as 1D NMR spectra.

The ^1H 1D projections of 2D J-resolved, virtually decoupled NMR spectra data processing was carried out using MestReNova 12.0.4 software's automatic phase and baseline correction. Calibration was made by using tetramethylsilane (δH = 0). Quantification of arginine, MMA, ADMA and SDMA used integration of characteristic peaks. Calculation of absolute concentrations is based on known concentrations of external standards. The ADMA levels relative to total amounts of arginine are calculated by the formula: ADMA/arginine (%) = (integrals (ADMA)/integrals (arginine)) * (integrals (100μM arginine)/integrals (100μM ADMA)), MMA/arginine (%) = (integrals (MMA)/integrals (arginine)) * (integrals (100μM arginine)/integrals (100μM MMA)).

High performance liquid chromatography (HPLC) assay

To verify the accuracy of NMR results, cell hydrolysates were compared with an established chromatographic method with slight modifications (Meinitzer et al., 2007). Samples were derivatized with an autosampler by mixing with o-phtalaldehyde solution (1mg/mL in 0.2 M borate puffer pH=9.5 with 0.5% mercaptopropionic acid). After a two-minute incubation, the mixture was injected. Arginine and metabolites were separated on a Chromolith® Performance RP-18e, column 100 x 4.6 mm (Merck, Darmstadt, Germany) with an isocratic mobile phase (flow 2.0 mL/min) consisting of 50 mM KH_2PO_4 pH=6.8 and 6% (v/v) acetonitrile. After 15 min, the column was regenerated for 2 min with a mixture of 50 mM KH_2PO_4 pH=6.5 and 50% (v/v) acetonitrile and reequilibrated before the next injection. The compounds were detected with a fluorescence detector (Agilent 1260 FLD, Santa Clara, CA, USA) at excitation 340 nm and emission 455 nm. Data were acquired on the Agilent Chemstation version B04.03. In contrast to the original protocol, no internal standard was used and the analysis was performed with the standard addition method. Defined concentrations

of Arginine, ADMA, SDMA and MMA were added to the hydrolysates and analysed without and with the addition of pure substance. From the differences, the concentrations of the initial concentrations were calculated.

RNA isolation, reverse transcription and real-time PCR

RNA was isolated using TRIsureTM following the manufacturer's guidelines (Meridian BioscienceTM, Cincinatti, OH). Then 2 μg of RNA were reverse transcribed with the High Capacity cDNA Reverse Transcription Kit (Applied Biosystems, Carlsbad, CA) and quantitative real-time PCR was performed using the Bio Rad C1000 TouchTM Thermal Cycler combined with CFX96 Real Time SystemTM (Bio Rad Laboratories, Hercules, CA). For expression analyses, 6 ng cDNA were analysed in duplicate and normalised to the expression of the housekeeping gene cyclophilin A. Expression profiles were determined using the $2^{-\Delta\Delta CT}$ method.

QUANTIFICATION AND STATISTICAL ANALYSIS

Data are presented as mean ± standard deviation (SD). Statistical differences among multiple groups (one-way ANOVA) are indicated by p values of < 0.05 (*), < 0.01 (**), < 0.001 (***) or < 0.0001 (****). Statistical analyses and graphs were generated using Graph Pad Prism 5.01. software (GraphPad Software, La Jolla, CA, USA).

Cell Reports Methods, Volume *1*

Supplemental information

Global analysis of protein arginine methylation

Fangrong Zhang, Jakob Kerbl-Knapp, Maria J. Rodriguez Colman, Andreas Meinitzer, Therese Macher, Nemanja Vujić, Sandra Fasching, Evelyne Jany-Luig, Melanie Korbelius, Katharina B. Kuentzel, Maximilian Mack, Alena Akhmetshina, Anita Pirchheim, Margret Paar, Beate Rinner, Gerd Hörl, Ernst Steyrer, Ulrich Stelzl, Boudewijn Burgering, Tobias Eisenberg, Brigitte Pertschy, Dagmar Kratky, and Tobias Madl

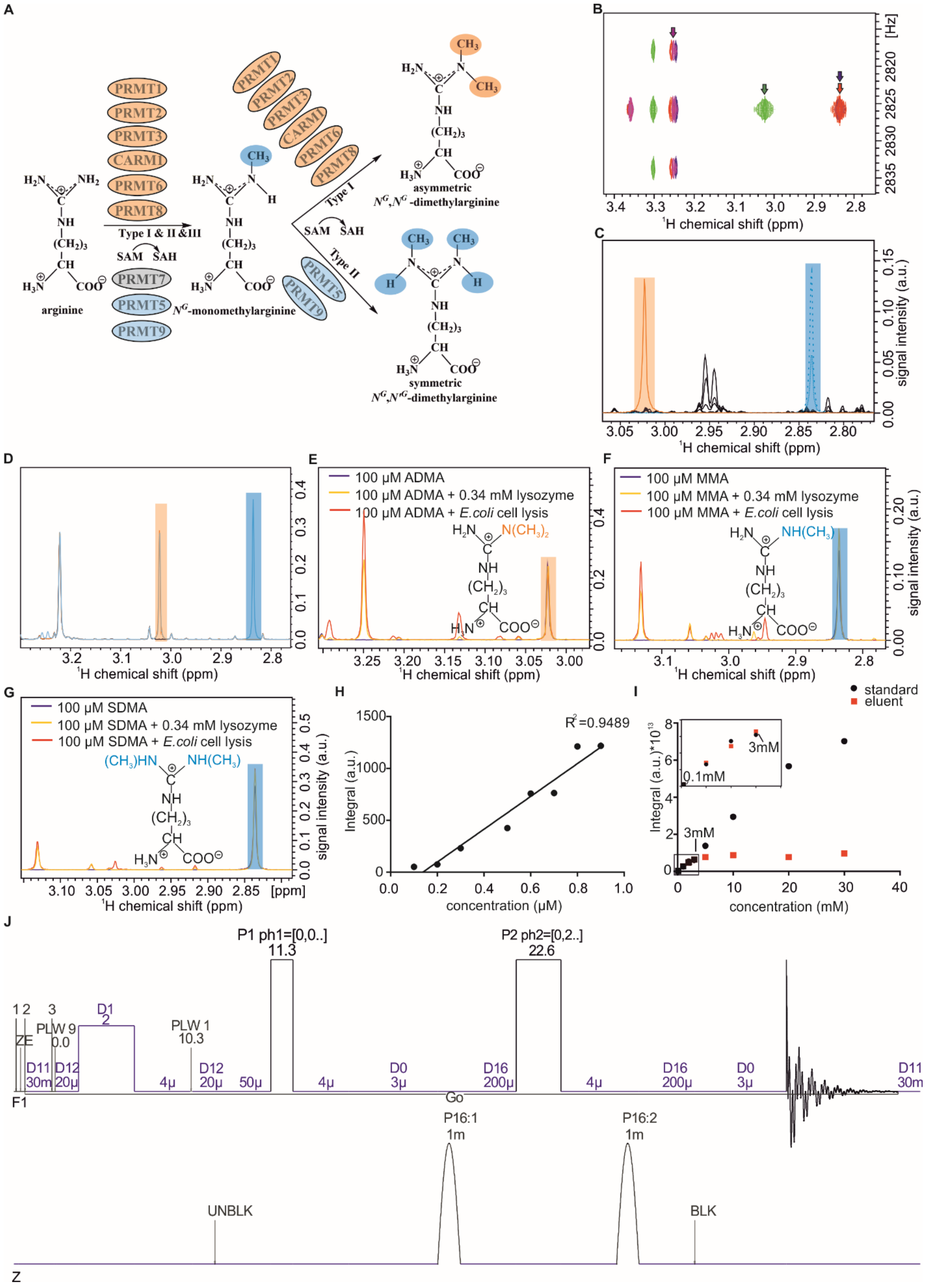
A
PRMT1
PRMT2
PRMT3
CARM1
PRMT6
PRMT8
Type I & II &III
SAM SAH
PRMT7
PRMT5
PRMT9
arginine
N^G-monomethylarginine
Type I
Type II
asymmetric
N^G,N^G-dimethylarginine
symmetric
N^G,N'^G-dimethylarginine
B
^{1}H chemical shift (ppm)
[Hz]
C
^{1}H chemical shift (ppm)
signal intensity (a.u.)
D
^{1}H chemical shift (ppm)
E
100 μM ADMA
100 μM ADMA + 0.34 mM lysozyme
100 μM ADMA + E.coli cell lysis
^{1}H chemical shift (ppm)
F
100 μM MMA
100 μM MMA + 0.34 mM lysozyme
100 μM MMA + E.coli cell lysis
^{1}H chemical shift (ppm)
signal intensity (a.u.)
G
100 μM SDMA
100 μM SDMA + 0.34 mM lysozyme
100 μM SDMA + E.coli cell lysis
^{1}H chemical shift (ppm)
[ppm]
signal intensity (a.u.)
H
R^2=0.9489
Integral (a.u.)
concentration (μM)
I
standard
eluent
Integral (a.u.)*10^{13}
3mM
0.1mM
concentration (mM)
J
P1 ph1=[0,0..]
11.3
P2 ph2=[0,2..]
22.6
ZE
PLW 9
0.0
D1
2
PLW 1
10.3
D11
30m
D12
20μ
4μ
50μ
D0
3μ
D16
200μ
Go
F1
P16:1
1m
P16:2
1m
UNBLK
BLK
Z

Figure S1. Related to Figure 1. Absolute quantification of protein arginine methylation by ArgMet-NMR.

(A) Arginine residues are methylated by the protein arginine methyltransferase (PRMT) (type I, orange; type II, blue; type III, grey) family members. PRMTs catalyze either the formation of monomethylarginine (MMA) (blue), asymmetric dimethylarginine (ADMA) (orange) or symmetric dimethylarginine (SDMA) (blue).
(B) Overlay of ^{1}H 2D J-resolved experiments of arginine (magenta), ADMA (green), MMA (purple) and SDMA (red), each 100 μM.
(C) Overlay of ^{1}H 1D projections of 2D J-resolved, virtually decoupled NMR spectra of *E. coli* lysates spiked with 100 μM ADMA, MMA and SDMA (black, solid line) before whole workflow, and 100 μM ADMA (orange, solid line), MMA (blue, dotted line) or SDMA (blue, dashed line), respectively, showing free methylarginines are negligible using our protocol.
(D) Overlay of 1H 1D projections of 2D J-resolved, virtually decoupled NMR spectra of metabolites extraction from HeLa cells (black), and then spiked with 100 μM ADMA (orange), MMA and SDMA (blue), respectively.
(E) Overlay of ^{1}H 1D projections of 2D J-resolved, virtually decoupled NMR spectra of ADMA recovery from lysozyme (yellow) and *E. coli* cell lysates (red). Shaded regions represent characteristic regions of ADMA (orange) methyl groups.
(F) Overlay of ^{1}H 1D projections of 2D J-resolved, virtually decoupled NMR spectra of MMA recovery from lysozyme (yellow) and *E. coli* cell lysates (red). Shaded regions represent characteristic regions of MMA (blue) methyl groups.
(G) Overlay of ^{1}H 1D projections of 2D J-resolved, virtually decoupled NMR spectra of SDMA recovery from lysozyme (yellow) and *E. coli* cell lysates (red). Shaded regions represent characteristic regions of SDMA (blue) methyl groups.
(H) Correlation between integrals and concentration changes of ADMA signal of ^{1}H 1D projections of 2D J-resolved spectra. Correlation coefficient (R^2 = 0. 9489, p = 0.033) was computed with the Pearson Product Moment statistic.
(I) Integrals of arginine (red, square) recovery from SPE compared to standard concentrations (black, circle).
(J) Pulse sequence of the 2D JRES NMR experiment.

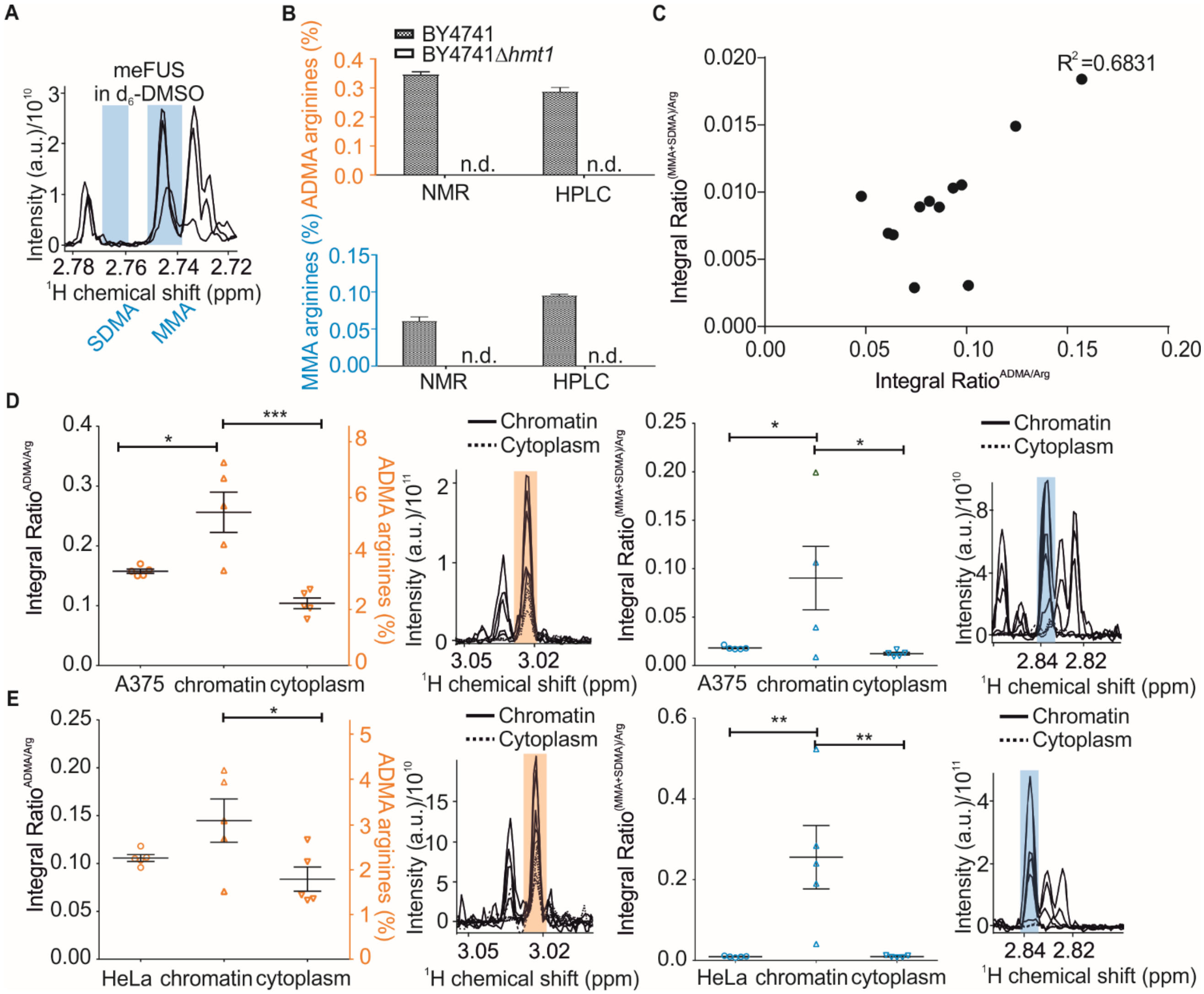

Figure S2. Related to Figure 2. Characterisation of ArgMet in purified proteins, yeast and mammalian cell lysates.

(A) Spectral overlays of characteristic MMA and SDMA NMR methyl signals in d_6-DMSO (n=3). Shaded regions represent characteristic regions of MMA and SDMA (blue) methyl groups.
(B) HPLC and NMR based protein ArgMet quantification of yeast lysates (n=3; mean ± SD; n.d. - not detectable). ADMA or MMA levels in relation to the total amount of arginine are indicated. The MMA peak obtained from HPLC showed spectral overlap interference.
(C) The correlation between integral ratios of ADMA/arginine and (SDMA/MMA)/arginine. Correlation coefficient (R^2 = 0. 6381, p = 0.0143) was computed with the Pearson Product Moment statistic.
(D) ArgMet quantification of chromatin and cytoplasm in A375 cells (n=5; mean ± SD; *p < 0.05, **p < 0.01, ***p < 0.001, ****p < 0.0001). Spectral overlays of characteristic ADMA (orange) and MMA/SDMA (blue), NMR methyl signals are shown as shaded regions.
(E) ArgMet quantification of chromatin and cytoplasm in HeLa cells (n=5; mean ± SD; *p < 0.05, **p < 0.01, ***p < 0.001, ****p < 0.0001). Spectral overlays of characteristic ADMA (orange) and MMA/SDMA (blue), NMR methyl signals are shown as shaded regions.

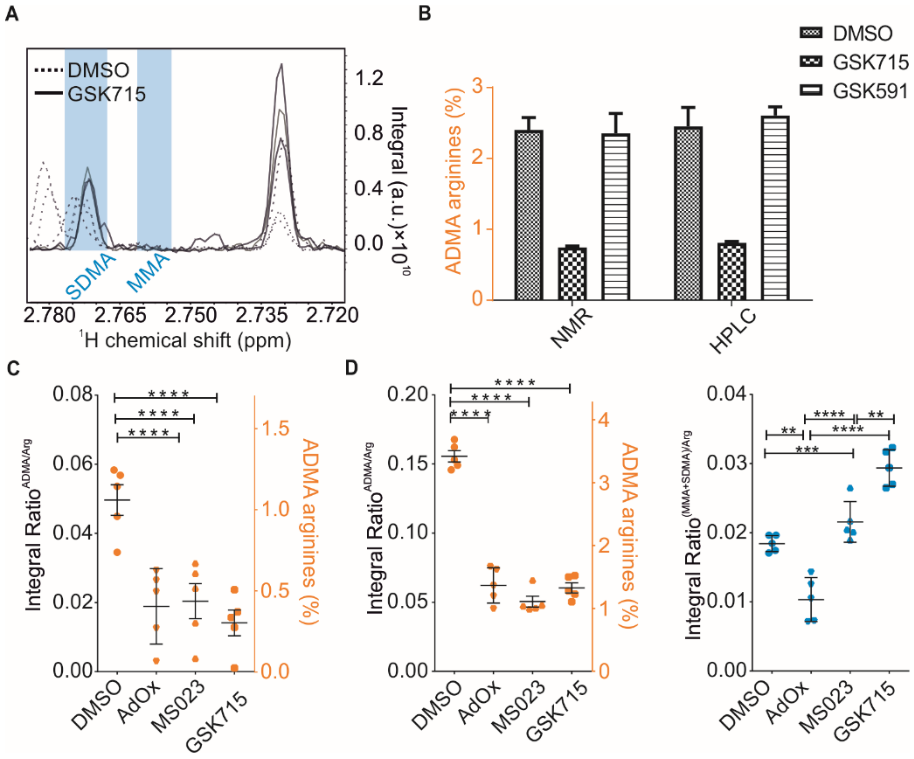

Figure S3. Related to Figure 3. ArgMet NMR enables quantification of protein ArgMet modulation and dynamics.

(A) Spectral overlays of characteristic MMA and SDMA NMR methyl signals in d_6-DMSO show that MMA and SDMA methyl resonances can be resolved (n=3), and higher SDMA represented in GSK715 than DMSO. Shaded regions represent characteristic regions of MMA and SDMA (blue) methyl groups.

(B) HPLC and NMR based protein ArgMet quantification of HeLa cells treated for 3 days with either DMSO, 2 µM GSK3368715 (GSK715) or 1 µM GSK3203591 (GSK591) (n=3; mean ± SD). ADMA levels in relation to the total amount of arginine are indicated.

(C) Protein ArgMet quantification of fibroblast cells treated for 3 days with either DMSO, 40 µM AdOx, 10 µM MS023, 2 µM GSK715 (n=5; mean ± SD; $*p < 0.05$, $**p < 0.01$, $***p < 0.001$, $****p < 0.0001$). ADMA levels with respect to total amounts of arginine are shown. Both MMA and SDMA are non-detectable in these conditions.

(D) Protein ArgMet quantification of A375 cells treated for 3 days with either DMSO, 40 µM AdOx, 10 µM MS023, 2 µM GSK715 (n=5; mean ± SD; $*p < 0.05$, $**p < 0.01$, $***p < 0.001$, $****p < 0.0001$). ADMA levels are presented with respect to total amounts of arginine.

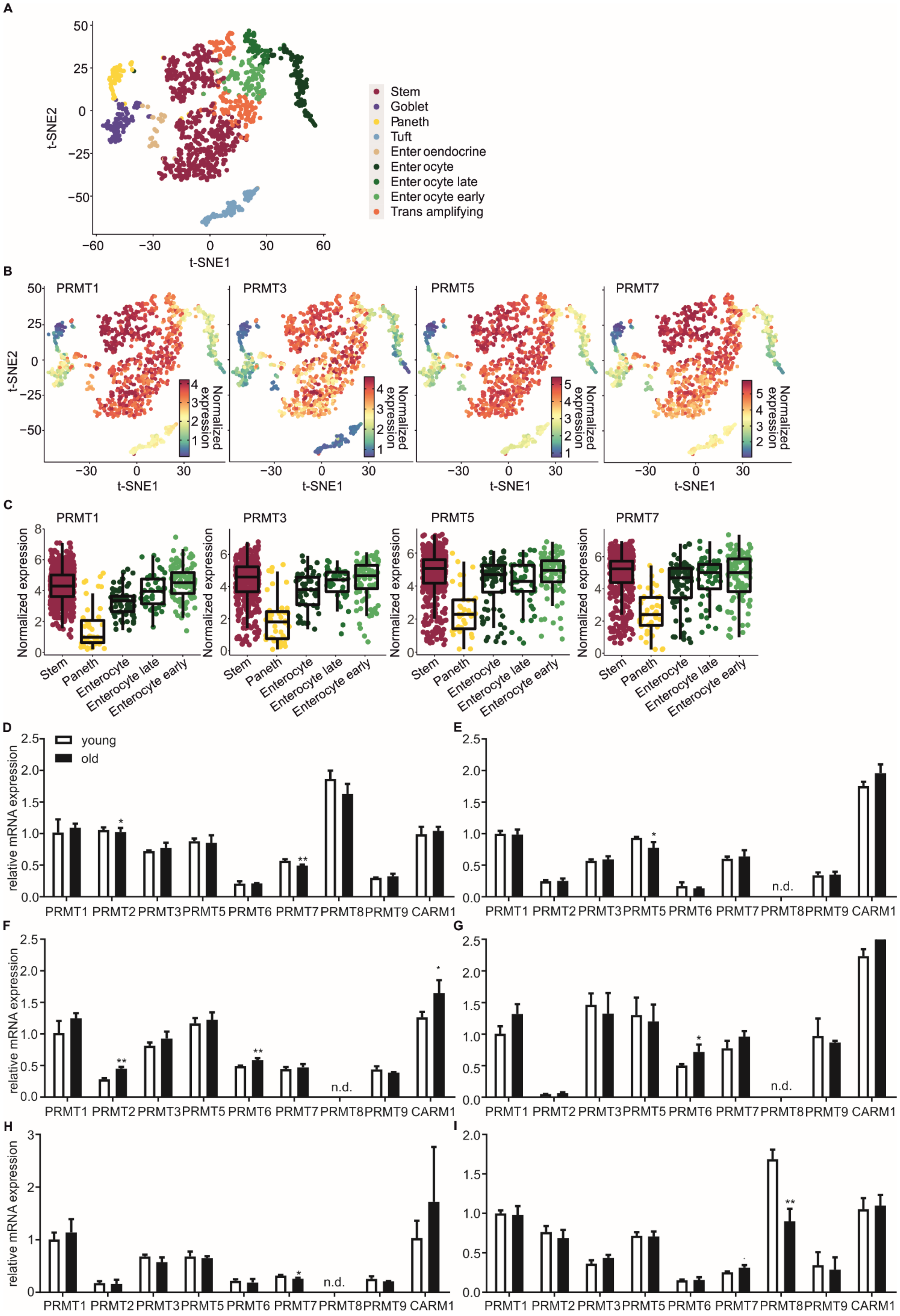

A
t-SNE2
t-SNE1
Stem
Goblet
Paneth
Tuft
Enter oendocrine
Enter ocyte
Enter ocyte late
Enter ocyte early
Trans amplifying
B
PRMT1
PRMT3
PRMT5
PRMT7
Normalized expression
C
Normalized expression
Stem
Paneth
Enterocyte
Enterocyte late
Enterocyte early
D
E
F
G
H
I
young
old
relative mRNA expression
PRMT1
PRMT2
PRMT3
PRMT5
PRMT6
PRMT7
PRMT8
PRMT9
CARM1
n.d.

Figure S4. Related to Figure 4. NMR enables characterization of ArgMet in cell differentiation and ageing *in vivo*.

(A) t-distributed stochastic neighbourhood embedding (t-SNE) visualization of 1,522 single-cell full-length sc-RNaseq data. Cluster annotation was done based on the expression of known cell type markers.
(B) PRMT1, 3, 5 and 7 gene expression levels are plotted on t-SNE plots.
(C) Boxplots represent the quantification of gene expression in each cluster gene (median values and 25th and 75th percentiles). The analysis was performed on mouse small intestine single cell RNAseq data set from Haber *et al.* (Haber et al., 2017) and the analysis was performed as described in Ludikhuize *et al.* (Ludikhuize et al., 2020).
(D-I) mRNA expression of PRMTs in (D) brain, (E) heart, (F) kidney, (G) liver, (H) spleen and (I) lung from young (9-11 weeks) and old mice (96-104 weeks) analysed by real-time PCR and normalized to cyclophilin A as reference gene. Expression profiles and associated statistical parameters were determined by the $2^{-\Delta\Delta Ct}$ method. Data represent means ± SD (n=3); *p < 0.05, **p < 0.01, ***p < 0.001.

**Table S1. List of oligonucleotides**		
Name	Sequence	Note
PRMT1 forward primer	GCCCGCAAGGTTATTGGGAT	real-time PCR
PRMT1 reverse primer	TGGTCTAACTTGTTGGCTTTGAC	real-time PCR
PRMT2 forward primer	AGCGCCGAGAAAGACTACC	real-time PCR
PRMT2 reverse primer	GGCCTTGAAAAGAACTCCTTGA	real-time PCR
PRMT3 forward primer	GGGATCGGTCTACCCTGACAT	real-time PCR
PRMT3 reverse primer	AGCTTCCGGTATAACTGCTTTC	real-time PCR
PRMT5 forward primer	GGAACTCTGAAGCGGCTATGT	real-time PCR
PRMT5 reverse primer	CTTCCTGATTAAGGGGCAATAGG	real-time PCR
PRMT6 forward primer	GATGGGCTACGGACTTCTGC	real-time PCR
PRMT6 reverse primer	GCATCTGGTCGCTAATCGGG	real-time PCR
PRMT7 forward primer	GCCAGGTCATCCTATGCCG	real-time PCR
PRMT7 reverse primer	GCCAATGTCAAGAACCAAGGC	real-time PCR
PRMT8 forward primer	ACGTGGTAGCAATCGAAGACA	real-time PCR
PRMT8 reverse primer	GCTCCTTCATGGCAACATCC	real-time PCR
PRMT9 forward primer	CAGAAACTGTCGATGCAGGTG	real-time PCR
PRMT9 reverse primer	AGCACCAACCCTATGATGCC	real-time PCR
CARM1 forward primer	ATCGCCCTCTACAGCCATGA	real-time PCR
CARM1 reverse primer	CTGTCTGCCCACACGACTG	real-time PCR

Table S1. Related to STAR Methods "RNA isolation, reverse transcription and real-time PCR" Key Resources Table subheading "Oligonucleotides". Shown are all oligonucleotides used in this study for real-time PCR.
